# A novel methodology for emotion recognition through 62-lead EEG signals: multilevel heterogeneous recurrence analysis

**DOI:** 10.3389/fphys.2024.1425582

**Published:** 2024-07-25

**Authors:** Yujie Wang, Cheng-Bang Chen, Toshihiro Imamura, Ignacio E. Tapia, Virend K. Somers, Phyllis C. Zee, Diane C. Lim

**Affiliations:** ^1^ Department of Industrial and Systems Engineering, University of Miami, Coral Gables, FL, United States; ^2^ Division of Sleep Medicine, Department of Medicine, University of Pennsylvania, Phialdelphia, PA, United States; ^3^ Division of Pulmonary and Sleep Medicine, Children’s Hospital of Philadelphia, Phialdelphia, PA, United States; ^4^ Division of Pediatric Pulmonology, Miller School of Medicine, University of Miami, Miami, FL, United States; ^5^ Department of Cardiovascular Medicine, Mayo Clinic, Rochester, MN, United States; ^6^ Center for Circadian and Sleep Medicine, Department of Neurology, Feinberg School of Medicine, Northwestern University, Chicago, IL, United States; ^7^ Department of Medicine, Miami VA Medical Center, Miami, FL, United States; ^8^ Department of Medicine, Miller School of Medicine, University of Miami, Miami, FL, United States

**Keywords:** heterogeneous recurrence analysis, emotion recognition, multi-channel EEG, dynamic system, ensemble learning

## Abstract

**Objective:**

Recognizing emotions from electroencephalography (EEG) signals is a challenging task due to the complex, nonlinear, and nonstationary characteristics of brain activity. Traditional methods often fail to capture these subtle dynamics, while deep learning approaches lack explainability. In this research, we introduce a novel three-phase methodology integrating manifold embedding, multilevel heterogeneous recurrence analysis (MHRA), and ensemble learning to address these limitations in EEG-based emotion recognition.

**Approach:**

The proposed methodology was evaluated using the SJTU-SEED IV database. We first applied uniform manifold approximation and projection (UMAP) for manifold embedding of the 62-lead EEG signals into a lower-dimensional space. We then developed MHRA to characterize the complex recurrence dynamics of brain activity across multiple transition levels. Finally, we employed tree-based ensemble learning methods to classify four emotions (neutral, sad, fear, happy) based on the extracted MHRA features.

**Main results:**

Our approach achieved high performance, with an accuracy of 0.7885 and an AUC of 0.7552, outperforming existing methods on the same dataset. Additionally, our methodology provided the most consistent recognition performance across different emotions. Sensitivity analysis revealed specific MHRA metrics that were strongly associated with each emotion, offering valuable insights into the underlying neural dynamics.

**Significance:**

This study presents a novel framework for EEG-based emotion recognition that effectively captures the complex nonlinear and nonstationary dynamics of brain activity while maintaining explainability. The proposed methodology offers significant potential for advancing our understanding of emotional processing and developing more reliable emotion recognition systems with broad applications in healthcare and beyond.

## 1 Introduction

The brain, one of the most intricate systems of the body, has been a subject of great interest for researchers aiming to unravel its complexities (Wolpaw and Birbaumer, 2006). The complexity of underlying nature (genetics) and the effect of nurture (life choices and experiences) creates an infinite number of possible stimuli and interactions, resulting in an evolving dynamic system within the brain. Understanding this dynamic system is crucial due to its pivotal role in various domains, including cognition, behavior, sleep, neurological disorders, and emotion (Lindquist et al., 2012; Akhand et al., 2023). To thoroughly explore this dynamic system, advanced technologies like functional magnetic resonance imaging (fMRI) and electroencephalography (EEG) have been employed to measure brain activity and study interactions with the environment (Jellinger, 2003; Haynes and Rees, 2006; Tong and Pratte, 2012). Recently, EEG has become available as a wearable technology, making it an ideal choice for continuous monitoring of neural processes and brain activity.

Emotions are complex psychophysiological processes, yet universally, they are experienced similarly by all people. Thus, the study of emotion recognition has garnered significant attention in various fields, such as neurology, computer science, cognitive science, and psychology (Lindquist et al., 2012; Akhand et al., 2023). Prior research has leveraged the time-domain, (Liu et al., 2021; Chen D. et al., 2023), frequency-domain, (Gao et al., 2019; Houssein et al., 2022; Akhand et al., 2023), or time-frequency domain methods (Yuvaraj et al., 2023) to extract the features within EEG signals to identify emotions. Recent research (Chang et al., 2022; Yang et al., 2022) has focused on leveraging artificial intelligence and neural network models to enhance the accuracy and efficiency of emotion classification based on EEG data (Li J. et al., 2021; Tian et al., 2021). Dan et al. introduced a clustering-promoting semi-supervised method to enhance the performance of emotion recognition (Dan et al., 2021). Wang et al. established a convolutional neural network (CNN) framework for emotion recognition (Wang et al., 2020). These advancements not only contributed to the field of neuroscience but also have practical applications in human-computer interaction and mental health diagnoses (Chai et al., 2018). Thus, EEG has become an important technology for objective emotion recognition (Peng et al., 2023).

Recent developments in EEG-based emotion recognition have focused on improving classification accuracy and robustness through various techniques such as feature fusion, dynamic functional connectivity analysis, and deep learning architectures. Fusing frequency-domain features and brain connectivity features has shown promising results in cross-subject emotion recognition (Chen et al., 2022a). Dynamic functional connectivity analysis has also been employed to capture the time-varying characteristics of brain networks during emotional states (Liu et al., 2019). Novel deep learning architectures, such as deep CNNs (Chen J. et al., 2019), multi-scale masked autoencoders (Pang et al., 2024), transformer- and attention-based CNNs (Li C. et al., 2021; Si et al., 2023) have been proposed to enhance emotion recognition performance. Domain adaptation techniques have also been explored to facilitate the transfer of emotion recognition models across different subjects (Chen et al., 2022b). In addition to emotion recognition, EEG-based approaches have been applied to related fields, such as P300 wave detection, driving fatigue detection, and biometric authentication, where self-attentive channel-connectivity capsule networks (Chen C. et al., 2023; Wang et al., 2023) and attention-based multiscale CNN with dynamical graph convolutional network (GCN) (Wang et al., 2021) have demonstrated improved performance. Systems like E-Key (Xu et al., 2023a) combine biometric authentication with driving fatigue detection. EEG studies have also examined the effects of aging, task difficulty, and training on working memory capacities, highlighting EEG’s diverse applications in cognitive research (Xu et al., 2023b).

Despite the progress made in EEG-based emotion recognition, several challenges remain. First, the nonlinear and nonstationary characteristics of EEG signals pose significant difficulties (Bazgir et al., 2018). Most machine learning based methodologies, such as linear discriminant analysis (Chen DW. et al., 2019), generalized linear regression (Li et al., 2019a), or Fast Fourier Transform (FFT) (Murugappan and Murugappan, 2013), often rely on linear assumptions, which fail to capture the nuanced nonlinear and nonstationary characteristics of EEG. Second, the complexity of multiple EEG electrodes capturing the interaction of brain activity and large volumes of data is another challenge. Deep learning models can address this complexity; however, they suffer from the “black box” problem while requiring substantial computational resources. Third, EEG signals present challenges in both temporal and spatial domains. While many studies focus on the temporal aspects of emotions (Liu et al., 2010; Zheng et al., 2019a), spatial information is equally important when adapting these methodologies in the future to neurological, sleep, or psychological disorders. Lastly, emotions are interconnected over time, with current emotional states being influenced by past emotions and potentially impacting future experiences (Thornton and Tamir, 2017). These transitions, between past, present, and future, have not been well studied using EEG signals.

To tackle these challenges, this paper presents an innovative three-phase methodology that characterizes and quantifies complex dynamic transitions of brain activities in multiple granularities while retaining high resolution to detect emotions from multi-channel EEG. In the first phase, manifold learning techniques are utilized to embed the dimensionality of high-dimensional 62-lead EEG signals into a more manageable lower-dimensional space. This embedding preserves the complex spatiotemporal characteristics of the signals, offering rich insights into brain activity while enhancing computational efficiency. In the second phase, we propose a novel multilevel heterogeneous recurrence analysis to characterize the nuanced, nonlinear, and nonstationary dynamic characteristics of the EEG signals at different granularities within the state-space domain. Our approach results in a quantification of dynamic patterns characterizing underlying brain activity, which cannot be achieved by other methods. The final phase employs ensemble supervised learning models that utilize metrics that quantify dynamic features and patterns within the EEG to classify each emotion. Ensemble learning not only improves overall performance but also provides a robust framework to prevent potential overfitting and account for variability in EEG data. This phase explains the decision-making processes underlying emotion classification. Experimental results show that our proposed methodology achieved accuracy and area under the receiver operating characteristic (ROC) curve (AUC) values of 0.7885 and 0.7552, respectively. These results surpass state-of-the-art studies using the same dataset. Moreover, our methodology provides the most consistent performance across different emotions compared to other models. Lastly, our method provides subtle quantifications and rich insights into the dynamic features of brain activity related to emotions.

In summary, this research introduces a novel recurrence analysis-based methodology for EEG-based emotion recognition that effectively captures the complex nonlinear and nonstationary dynamics of brain activity while maintaining explainability. The rest of this paper is organized as follows: Section 2 is a brief background relevant to our methodology; Section 3 describes the dataset employed to formulate our approach; Section 4 outlines the proposed methodology, structured in three distinct phases; Section 5 details the outcomes of our study; and Section 6 offers an in-depth discussion of the insights gained and conclusions drawn from our investigation.

## 2 Research background

In this section, we introduce the foundational concepts and background of our novel methodology, multilevel heterogeneous recurrence analysis (MHRA). We begin by discussing the basic principles of recurrence analysis (RA) and its evolution into heterogeneous recurrence analysis (HRA). Then, we review the development and application of HRA to complex transitions, which is further developed and refined into MHRA.

### 2.1 Recurrence analysis

Recurrence, defined as a situation where the state of a system at a certain time is very similar to its state at one or more previous times, is a fundamental feature of complex systems (Hatami et al., 2019). From Poincaré's initial descriptions of recurrence in the 1890s and the subsequent introduction of Recurrence Analysis (RA) by Webber and Zbilut in the 1980s (Khoo et al., 1996), the development of this analytical method has continuously evolved. In the early 2000s, Norbert Marwan and his colleagues made significant contributions to refining and applying RA, thereby enhancing its use across a variety of scientific fields, including geophysics (Eroglu et al., 2014; Lucarini et al., 2016), physiology (Khoo et al., 1996; Webber and Zbilut, 2005), meteorology (Bouabdelli et al., 2020), economics (Mosavi et al., 2020), and engineering (Shu et al., 2021). Consequently, RA has become one of the most widely used tools for analyzing dynamic complex systems. Note that the recurrence can be mathematically defined as $R_{i,j}$ in Eq. 1, indicating whether a recurrence exists between system states $s_{i}$ and $s_{j}$ . If the proximity of $s_{i}$ and $s_{j}$ , measured by $\left\|s_{i}-s_{j}\right\|$ , is smaller than a predefined threshold ϵ , then a recurrence exists between $s_{i}$ and $s_{j}$ (Eckmann et al., 1987; Marwan et al., 2007a; Marwan, 2008).

$$R_{i,j}=H\left(\epsilon -\left\|s_{i}-s_{j}\right\|\right)$$ (1)

where $H\left(x\right)$ is a Heaviside function, in which $H\left(x\right)=1$ if x≥0 , and $H\left(x\right)=0$ otherwise; (Eckmann et al., 1987) $s_{t}$ is the system state at time t . The recurrence of the system over a period of observation window is then represented as a symmetric matrix $R=\left\{R_{ij},\forall i,j\right\}$ , which can be geometrically visualized as a Recurrence Plot (RP), typically shown as a dot plot where each axis represents the entire observation period and a dot plotted in the coordinate ( i , j ) indicates a recurrence exists between time i and j . This visualization not only highlights the frequency of recurrence but also reveals patterns and structures indicative of the dynamical behavior of the system, such as stability, periodicity, or chaotic dynamics (Eckmann et al., 1987). With analyzing the sophisticated geometric patterns in the RP, the nonlinear, nonstationary, and dynamic system characteristics are then quantified and characterized, known as Recurrence Quantification Analysis (Webber and Zbilut, 2005; Webber and Marwan, 2015). Notably, Marwan et al. generalized RP from a two dimensional matrix to a four dimensional tensor to capture the recurrence patterns within spatial data (Marwan et al., 2007b). RA has achieved tremendous success in various fields, for instance, it has been used to improve the normalization of electromyography signals (Avdan et al., 2023) detect series arc faults in photovoltaic systems (Amiri et al., 2022) and analyze histopathological images (Wang and Chen, 2022). Additionally, Donner et al. leveraged network topology to interpret the recurrence matrix **R**, thereby developing a novel analytical framework known as the recurrence network (RN). This approach provides another perspective for effectively parsing the dynamic features of complex systems (Donner et al., 2010; Donner et al., 2011). Notably, our previous work developed an innovative RN to analyze the complex patterns in spatial data, which has already been implemented in characterizing surface roughness in ultra-precision machining (Chen et al., 2018) and in detecting invasive ductal carcinoma in breast cancer (Chen CB. et al., 2023).

### 2.2 Heterogeneous recurrence analysis

Traditional RA, including RP and RN, treats recurrence homogeneously, which presents limitations when characterizing nuanced dynamic features. To improve RA, Yang et al. developed HRA, which addresses the heterogeneity of recurrence and dramatically enhances the analytical capabilities (Yang and Chen, 2014; Chen and Yang, 2015; Chen and Yang, 2016). HRA differentiates recurrences based on the properties of system states, categorizing each state $s_{t}$ into K different groups, denoted as $L\left(s_{t}\right)=k\in \left\{1,2,\ldots ,K\right\}$ for all t . It is crucial to note that the states within one category share similar system properties, while states in different categories exhibit distinct system properties. Heterogeneous recurrence is mathematically represented as Eq. 2:

$$\Omega_{ij}=L\left(s_{i}\right)\cdot H\left(0-\left\|L\left(s_{i}\right)-L\left(s_{j}\right)\right\|\right)$$ (2)

where $L\left(s_{t}\right)$ indicates the category of state $s_{t}$ for all t , $\left\|\cdot \right\|$ represents the norm, and $H\left(\cdot \right)$ denotes a Heaviside function. This approach means that if $s_{i}$ and $s_{j}$ belong to the same category $L\left(s_{t}\right)$ , a recurrence exists between $s_{i}$ and $s_{j}$ in category $L\left(s_{i}\right)$ . This method not only enhances the resolution of single-state recurrences but also reveals the sophisticated dynamics of transitions, which are often limited by conventional RA. Furthermore, HRA employs the Iterated Function System (IFS), an iterative projection function used to construct fractals, to project a sequence of transitions into a fractal space. This utilization of a fractal structure’s geometric features allows for a detailed characterization of complex dynamic properties associated with transitions (Yang and Chen, 2014). The analysis and quantification of these geometric structures, termed Heterogeneous Recurrence Quantification Analysis (HRQA), enable HRA to provide greater resolution in characterizing complex dynamic patterns. HRA has been successfully implemented to characterize complex systems in various fields, including finance (Zhang et al., 2023) medicine (Chen and Yang, 2015; Chen and Yang, 2016; Cheng et al., 2016; Chen et al., 2020; Avdan et al., 2024) physics (Yang and Chen, 2014) and engineering (Kan et al., 2016; Yang et al., 2020; Peng and Chen, 2023). Notably, Chen et al. extended the HRA to develop Spatial HRA (SHRA) for investigating complex recurrence patterns in spatial data. SHRA has been implemented in medical imaging (Yang et al., 2020; Van Booven et al., 2024a; Van Booven et al., 2024b) and additive manufacturing (Chen R. et al., 2019; Chen, 2019). However, while HRA can effectively characterize subtle nonlinear dynamic properties including complex transitions of a system, there has been little development of systematically investigating system dynamics across multiple scales, which could reveal additional system characteristics (Chen et al., 2017; Chen C-B. et al., 2019). To address this gap, we developed a novel HRA-based methodology to more precisely define multilevel transitions.

## 3 Data: 62-lead EEG signals

We utilized the Shanghai Jiao Tong University (SJTU) Emotion EEG Dataset for Four Emotions (SEED-IV), a specific subset of the broader SJTU Emotion EEG Dataset (available at https://bcmi.sjtu.edu.cn/∼seed/), to develop our methodology for emotion recognition (Zheng et al., 2019b). The SEED-IV dataset includes both EEG and eye movement signals associated with four distinct emotions, neutral, sadness, fear, and happiness, collected from 15 college-aged participants (seven males and eight females, aged 20–24, all right-handed). Each participant was outfitted with a 62-channel EEG cap (Compumedics Neuroscan, Australia) and eye-tracking glasses (SensoMotoric Instruments, Germany). The data were gathered while participants watched 72 carefully selected film clips, each designed to elicit one of the target emotions. Each clip had a duration of approximately 2 minutes and was shown only once to avoid the effects of repetition. Participants attended three separate sessions on different days, each comprising 24 trials with six trials per emotion. Each trial began with a 5-s introductory hint, followed by a 45-s period for self-assessment, during which participants rated their emotional experience. Data from participants who either did not experience the intended emotion or exhibited weak emotional arousal were excluded from the analysis. The primary objective of this research is to identify these four emotions using dynamic features extracted from multi-channel EEG signals. For the purposes of this study, we focused exclusively on the raw EEG data from 62 channels, capturing the complex brain dynamics associated with each emotional state, while the eye movement data were not utilized in the analysis.

## 4 Multilevel heterogeneous recurrence analysis for emotion recognition

**FIGURE 1:**
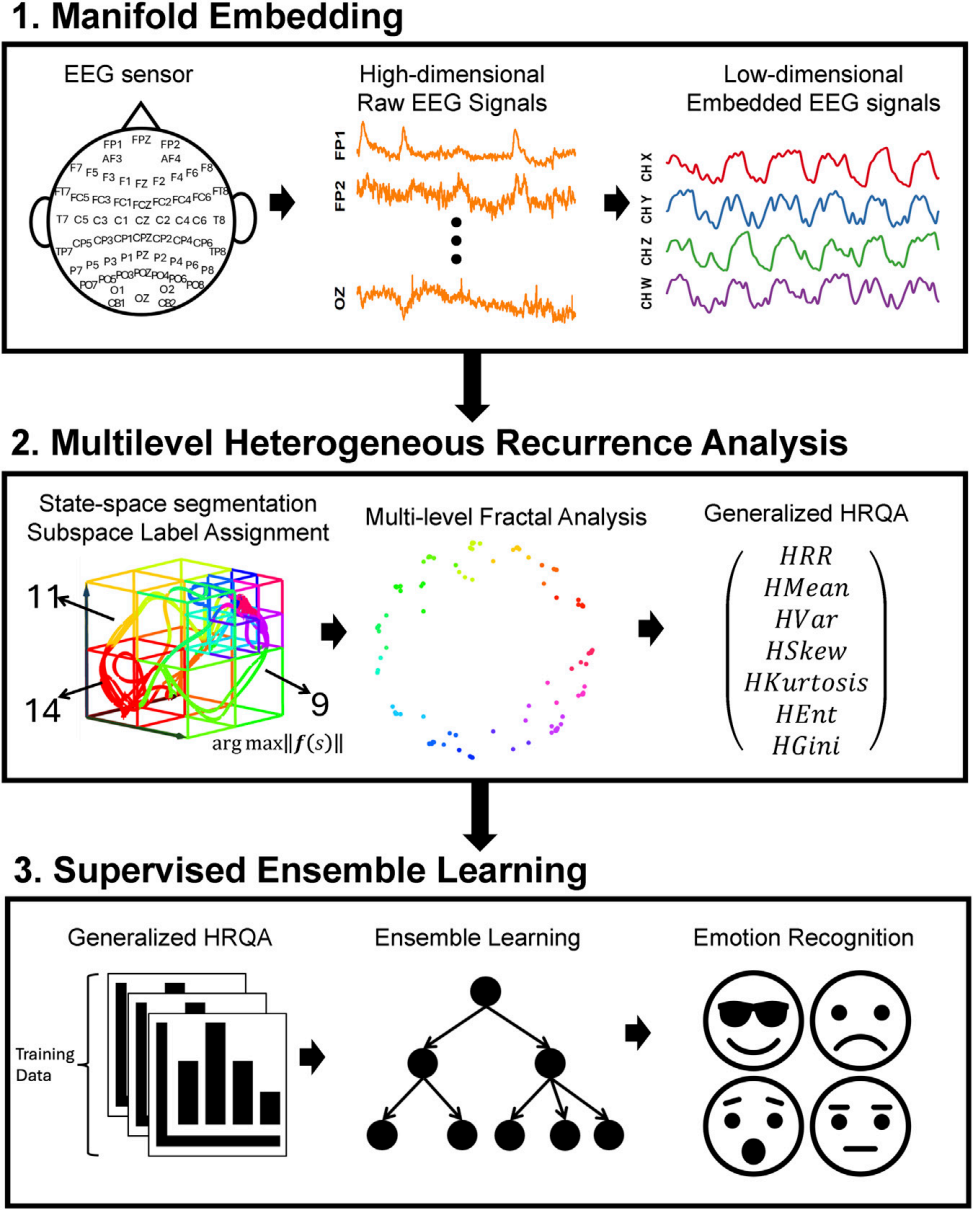
Overview of three-phase methodology, MHRA methodology, applied to EEG for emotion recognition. Phase 1. Manifold Embedding: A manifold learning method is applied to high-dimension EEG data to embed subtle nonlinear spatiotemporal characteristics into lower dimensions, reducing computational demands. Phase 2. MHRA: We developed the MHRA to quantify dynamic transitions using fractal representation at multiple levels. Phase 3. Supervised Ensemble Learning: Advanced ensemble learning methods are leveraged to analyze MHRA metrics for emotion recognition.

This study aims to identify four emotions by analyzing the complex spatiotemporal dynamics within high-dimensional EEG signals. We developed a novel three-phase methodology, named MHRA methodology, summarized in Figure 1, to accomplish this goal. The methodology comprises the following phases: Phase 1. Manifold Embedding: To preserve the intricate nonlinear spatiotemporal characteristics of raw EEG data while minimizing computational demands, we employed a manifold learning technique. This method projects the high-dimensional EEG data into a lower-dimensional space, thereby simplifying the dataset while retaining its essential features. Phase 2. MHRA: To capture the complex dynamic brain activity reflected in EEG signals, we developed a novel MHRA. This approach systematically portrays the multilevel dynamic characteristics of EEG data using fractal structures and quantifies the geometric features of these fractals to extract dynamic features for emotion recognition. Phase 3, Supervised Ensemble Learning: To differentiate emotions based on the dynamic properties extracted from EEG signals, we utilized various advanced ensemble learning techniques, including Random Forest, XGBoost, and Adaboost. The high accuracy achieved by our proposed model highlights the crucial role these dynamic properties play in effectively recognizing emotions. Further details of each phase are discussed in the remainder of this section.

### 4.1 Phase 1: manifold embedding

Massive data sizes and high dimensionality are two notorious obstacles in the field of data analytics. Effectively retaining data properties while efficiently processing data is crucial. This study analyzes data from 62-lead EEG signals, which presents significant challenges due to their massive data size and high dimensionality. Although these high-dimensional data offer superior spatiotemporal resolution, the inherent complexities of these EEG signals significantly increase the difficulties of data processing and analysis. Particularly in terms of the highly computational demands they impose. Therefore, reducing analytical and computational efforts to a manageable level while retaining the original data’s spatiotemporal characteristics is essential. Traditional dimensionality reduction techniques, such as principal component analysis and singular value decomposition, often fall short with large, complex datasets. They tend to overlook the nonstationary, nonlinear features of the data, leading to extended computation times and ineffective dimension reduction outcomes that do not accurately reflect the original data’s information (Roweis and Saul, 1979; Elgamal and Hefeeda, 2015; Pouyet et al., 2018).

To address these challenges, we have utilized manifold embedding, a technique within manifold learning that is particularly effective at uncovering the low-dimensional manifold structure embedded in high-dimensional spaces. It allows us to map high-dimensional data onto a lower-dimensional space efficiently, retaining the data’s intrinsic and nonlinear properties. This simplification of the dataset preserves essential spatiotemporal information, facilitating further analysis (Turchetti and Falaschetti, 2019). Notably, manifold embedding encompasses various techniques collectively known as Nonlinear Dimensionality Reduction (NLDR). Common methods within NLDR include Uniform Manifold Approximation and Projection (UMAP), which constructs a high-dimensional graph representation of the data and then optimizes a low-dimensional graph to be as structurally similar as possible; Locally Linear Embedding (LLE), which preserves local properties of the data; Spectral Embedding, which uses the eigenvalues of the graph Laplacian to perform dimensionality reduction; Isomap, which preserves geodesic distances between data points; and t-distributed Stochastic Neighbor Embedding (t-SNE), which minimizes the divergence between two distributions: a distribution that measures pairwise similarities of the input objects and a distribution that measures pairwise similarities of the corresponding low-dimensional points in the embedding (Meilă and Zhang, 2024).

To select the most appropriate NLDR method, we consider both the quality of dimensional reduction and computational efficiency. For assessing reduction quality, we utilize cross-entropy to compare the differences between the original and reduced signals. Cross-entropy is expressed as Eq. 3:

$$C\left(l\right)=-\sum_{t}\left\|l\left(s_{t}\right)\right\|\log\left(\left\|s_{t}\right\|\right)\;$$ (3)

where $l\left(s_{t}\right)$ is the lower-dimensional projection of signals $s_{t}$ converted by function $l\left(\cdot \right)$ , and $\left\|\cdot \right\|$ takes $L_{2}$ -norm of multi-channel signals. The NLDR technique with the best retention of original signals within the reduced signals will have the lowest cross-entropy value, indicating they contain a similar amount of information.

**FIGURE 2:**
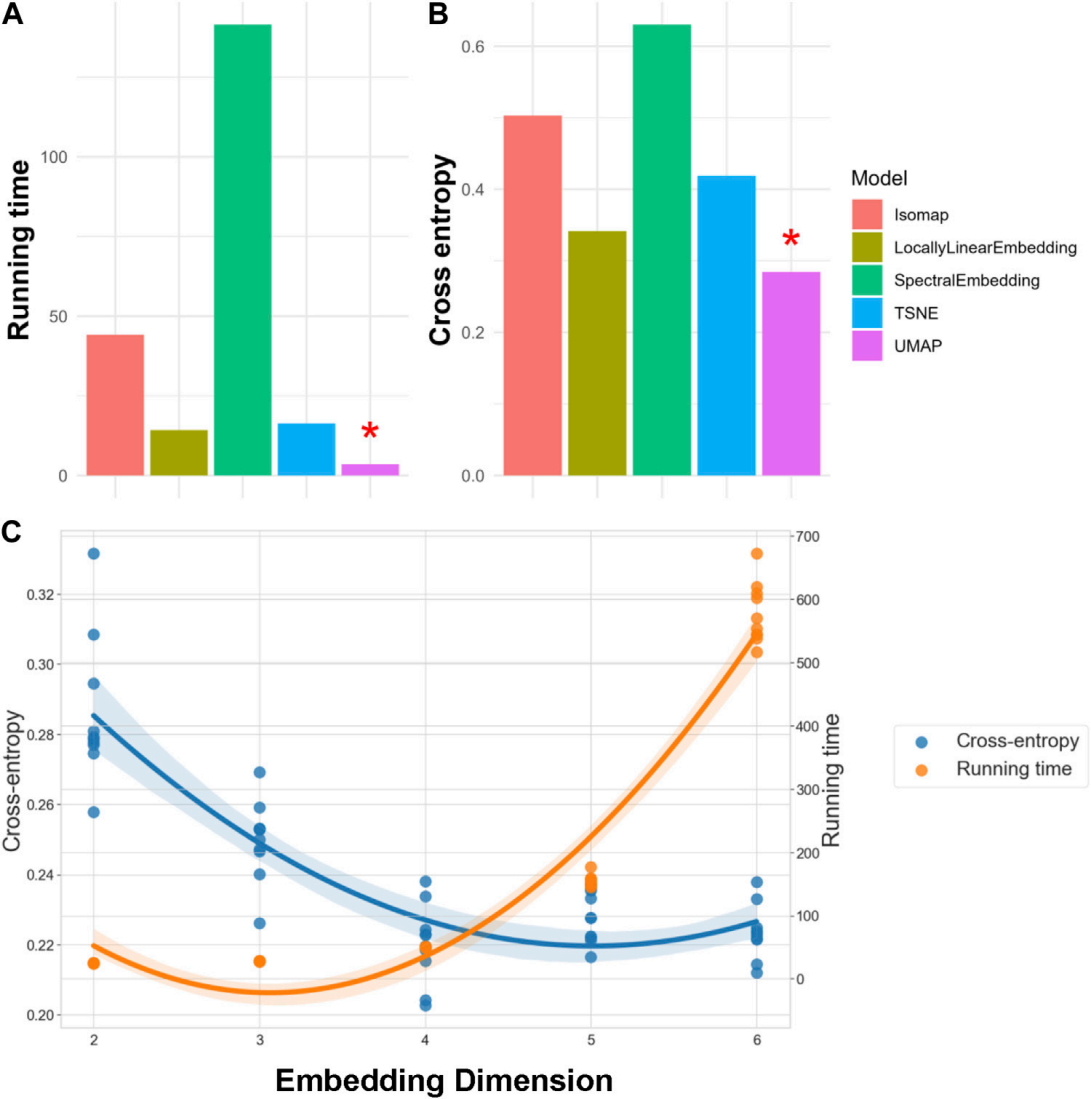
Evaluation of NLDR Methods and selecting the optimal number of Embedding Dimension. Panels **(A, B)** compare five manifold embedding candidates by running time and cross-entropy, respectively, indicating that UMAP is the best method for our specific dataset. Panel **(C)** illustrates how running time and cross-entropy were used to identify four as the optimal number of embedding dimensions to preserve critical spatiotemporal features within the dataset.

We evaluated each NLDR technique by analyzing a 10% random sample of SEED-IV data across ten replications. The performance of these manifold embeddings is presented in Figure 2. Panel A displays the average running time, while Panel B shows the average cross-entropy. Note that a lower running time indicates better efficiency, and a lower cross-entropy signifies higher information retention. For our 62-lead EEG data, UMAP not only achieved the lowest cross-entropy but also the best performance in terms of running time (Mcinnes et al., 2020), as indicated by a red asterisk. We used the same criteria, running time and cross-entropy, to determine the optimal number of embedding dimensions, referring to the number of dimensions in the lower-dimensional space. Our findings reveal that as embedding dimensions increase, the running time grows exponentially, while the improvement in cross-entropy diminishes. Figure C demonstrates these trends in UMAP, and it shows that the optimal performance, both in terms of running time and cross-entropy, occurs at four embedding dimensions. Notably, we also fine-tuned hyperparameters for all the manifold learning methods to optimize embedding performance. For our final selected method, UMAP, these hyperparameters included the number of neighbors (set to 5), the minimum distance between points in the low-dimensional space (set to 0.1), and the spread of the data points (set to 1.0). These settings were chosen to balance the retention of the data’s intrinsic structure and computational efficiency. Consequently, UMAP was selected to embed the 62-lead EEG signals into four dimensions, effectively balancing critical spatiotemporal feature retention with computational efficiency.

### 4.2 Phase 2: multilevel heterogeneous recurrence analysis

After embedding the 62-lead EEG signals into a low-dimensional space, we deployed the proposed MHRA to characterize the dynamic spatiotemporal characteristics of brain activity. The MHRA is a state-space domain method comprising three major steps: 1. Heterogeneous state-space representation, 2. Fractal representation, and 3. Generalized HRQA. These steps outline a systematic and comprehensive approach to characterizing complex dynamic systems.

#### 4.2.1 Heterogeneous state-space representation

To capture and delineate the recurrence dynamics of a system, we first transform time series data into a trajectory within a state space, S , representing all possible states of the system. Notably, each point of a *d*-dimensional time series is projected as a corresponding point in the *d*-dimensional state-space, denoted by $s_{t}=\left(x_{t}^{1},x_{t}^{2},\ldots ,x_{t}^{d}\right)\in S$ , where each dimension of the state space corresponds to a different measure of the system. Consequently, the evolution of the time series data forms a trajectory in this space, denoted as $s=\left\{s_{1},s_{2},\ldots ,s_{t}\right\}$ , and the geometric properties of this trajectory reveal the dynamic characteristics of the system.

**FIGURE 3:**
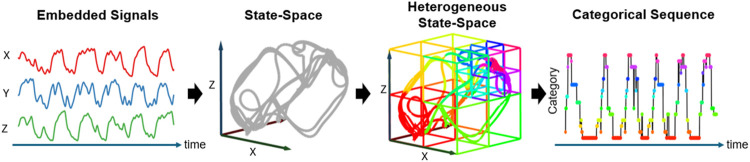
Heterogeneous State-Space Representation. This flowchart illustrates how EEG signals are transformed into a trajectory within the heterogeneous state space, and how these transitions are categorized into a dynamic sequence. The EEG signals are first transformed into a trajectory within the state space, followed by the application of Voronoi tessellation to segment the space into distinct subspaces. Each subspace, represented as a Voronoi cell, is assigned a specific category, illustrating the formation of a categorical sequence that captures the dynamic evolution of brain activity.

Subsequently, to achieve a higher resolution of the recurrence properties, we constructed a heterogeneous state-space by dividing the original state-space, S, into K subspaces, $S_{k},$ denoted as $S={⋃}_{k\in \left\{1,\ldots ,K\right\}}S_{k}$ . This segmentation helps differentiate recurrences, as system states within the same subspace exhibit similar system properties, and states in different subspaces display distinctly different system properties. Notably, there are many space segmentation methods that serve different purposes. This study utilizes one of widely used space segmentation method, Voronoi tessellation (Asghar et al., 2020), focusing on the similarity within each subspace when segmenting heterogeneous state-spaces. Therefore, by assigning the system states within the same subspace the same category label, denoted as $L\left(s_{t}\right)=k,{\forall s}_{t}\in S_{k},\forall k\in K=\left\{1,\ldots ,K\right\}$ , where L is a label assignment function maps each state $s_{t}$ to a categorical variable *k*, the trajectory of evolution forms a categorical sequence that reveals the dynamic transitions within the system. To ensure that the trajectory retains sufficient patterns to accurately represent sophisticated emotions, a 20-s window was employed to capture the characteristics of brain activity in this study. Figure 3 conceptually illustrates the process of heterogeneous state-space representation used in this study. Initially, the embedded EEG signals are transformed into a trajectory within the state space (shown in three dimensions for better visualization). Subsequently, a space segmentation method, Voronoi tessellation, is employed to create a heterogeneous state-space representation, where each Voronoi cell represents a distinct subspace. By assigning a category to each subspace, the EEG signals are converted into a categorical sequence that reveals the dynamic evolution of brain activity.

**FIGURE 4:**
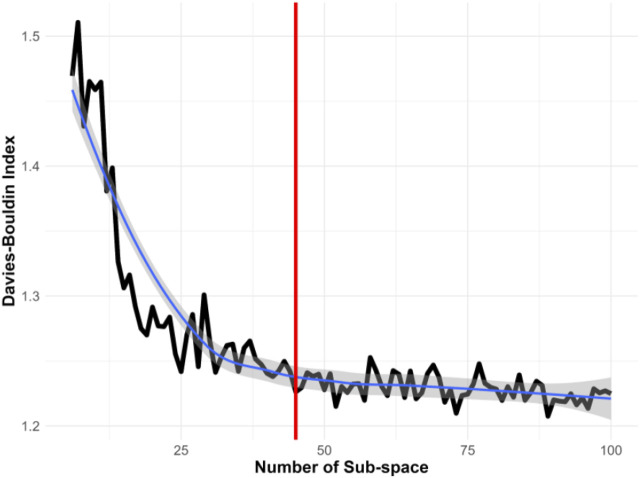
Determining the Optimal Number of Subspaces Using the Davies-Bouldin Index. This index assesses the effectiveness of different subspace configurations, with a lower score indicating better clustering quality. The analysis suggests that 45 subspaces provide the most informative clustering in this study.

Notably, Voronoi tessellation, typically a semi-supervised method, requires specifying the number of subspaces in advance. Selecting an inappropriate number of subspaces can significantly impact the effectiveness of information extraction. Determining the optimal number of subspaces is thus crucial for accurately representing the heterogeneous state-space. This research utilized the Davies-Bouldin Index, a measure of clustering quality, to find the optimal number of subspaces. Initially, as illustrated in Figure 4, we divided the original state-space into 10 subspaces and incrementally evaluated up to 100 subspaces. The black line represents the Davies-Bouldin Index, the smooth blue line indicates a fitted curve of the index values, and the grey shading denotes the confidence interval. A lower Davies-Bouldin Index indicates more effective clustering, with clear separation between subspaces. The index stabilized after 45 subspaces, identifying this number as optimal for our dataset. Accordingly, we segmented the state-space into 45 distinct subspaces to enhance the resolution of dynamic characteristics.

#### 4.2.2 Fractal representation

To characterize the dynamic characteristics of state transition patterns, this study leverages the fractal topological structure to capture the nuanced features. Fractals are mathematical structures portrayed by self-similarity, meaning each part of the fractal replicates the whole on a smaller scale. This intrinsic property makes fractals particularly suited for modeling heterogeneous recurrences, as their recursive nature can effectively mirror the irregular and complex patterns observed in such phenomena. By employing fractals, one can capture the nuanced nonlinear and nonstationary variations inherent in heterogeneous recurrences, providing a more accurate and comprehensive understanding of their dynamics (Yang and Chen, 2014; Cheng et al., 2016; Kan et al., 2016; Yang et al., 2020).

**FIGURE 5:**
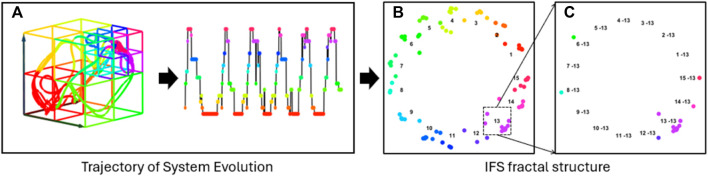
Fractal Structure Construction. Panel **(A)** displays the trajectory of system evolution as a categorical sequence, and **(B)** illustrates the projection of this trajectory into a unique fractal structure using an Iterated Function System (IFS). The self-similar nature of the fractal enables the investigation of dynamic patterns across multiple scales. **(C)** Depicts a second-level fractal derived from **(B)**, revealing dynamic characteristics on a different scale.

Therefore, after the embedded EEG signals are converted into a trajectory in the heterogeneous state space, revealing the system’s evolution, the trajectory is then projected into a fractal space using Iterated Function System (IFS). Notably, this IFS projection is a one-to-one mapping where each trajectory forms its own fractal structure that reveals the nuanced recurrence dynamics (as shown in Figure 5). Each transformed point strategically captures its transition order prior to its corresponding point in the state sequence.

The IFS iteratively maps each element of categorical sequence, k , which reflects the category of subspace of the corresponding embedded EEG signals, $L\left(s_{t}\right)=k\in K$ , to a unique IFS address in the fractal circle through the following function (Eq. 4):

$$I\left(t\right)=\left(\begin{array}{c} I_{x}\left(t\right)\\ I_{y}\left(t\right) \end{array}\right)=\Phi \left(k,\left(\begin{array}{c} I_{x}\left(t-1\right)\\ I_{y}\left(t-1\right) \end{array}\right)\right)=\left(\begin{array}{cc} \alpha & 0\\ 0 & \alpha \end{array}\right)\cdot \left(\begin{array}{c} I_{x}\left(t-1\right)\\ I_{y}\left(t-1\right) \end{array}\right)+\left(\begin{array}{c} \cos\left(\frac{2\pi k}{K}\right)\\ \sin\left(\frac{2\pi k}{K}\right) \end{array}\right),\text{with }I\left(0\right)=\left(\begin{array}{c} 0\\ 0 \end{array}\right)$$ (4)

where $\Phi \;\left(k,I\left(t-1\right)\right)$ maps an IFS address $I\left(t\right)$ based on the subspace category k at time t and incorporates the influence of all previous states provided by $I\left(t-1\right).$ The circular address is determined by two components: (1) current state and its assigned category variable *k*, via the transformation ${\left(\cos\left(2\pi k/K\right),\sin\left(2\pi k/K\right)\right)}^{T}$ ; (2) all the previous states, adjusted by a scaling factor α , through the iterative function. Note that α is defined as $\alpha =\tau \cdot \sin\left(\pi /K\right)/\left(1+\sin\left(\pi /K\right)\right)$ to ensure address remains distinct, where 0<τ<1 (in this study, τ=.99 ).

This IFS is designed to provide a self-similar fractal structure that embeds the information from all previous states, thereby enabling the formation of fractal patterns of spatial transitions at multiple scales. Note that this fractal structure allows us to investigate dynamic characteristics of transitions at multiple levels. For instance, as shown in Figure 5B, the distribution of 15 individual subspaces, $\left\{1,2,\ldots ,15\right\},$ shows the recurrence variations in different subspaces, named first-level transition; Figure 5C reveals the recurrence variations of two-state transitions, $\left\{\left\{1\to 13\right\},\left\{2\to 13\right\},\ldots ,\left\{13\to 13\right\}\right\}$ , named second-level transition, in a zoomed-in fractal of Figure 5B. This fractal representation precisely captures the nuanced characteristics of system dynamics.

**FIGURE 6:**
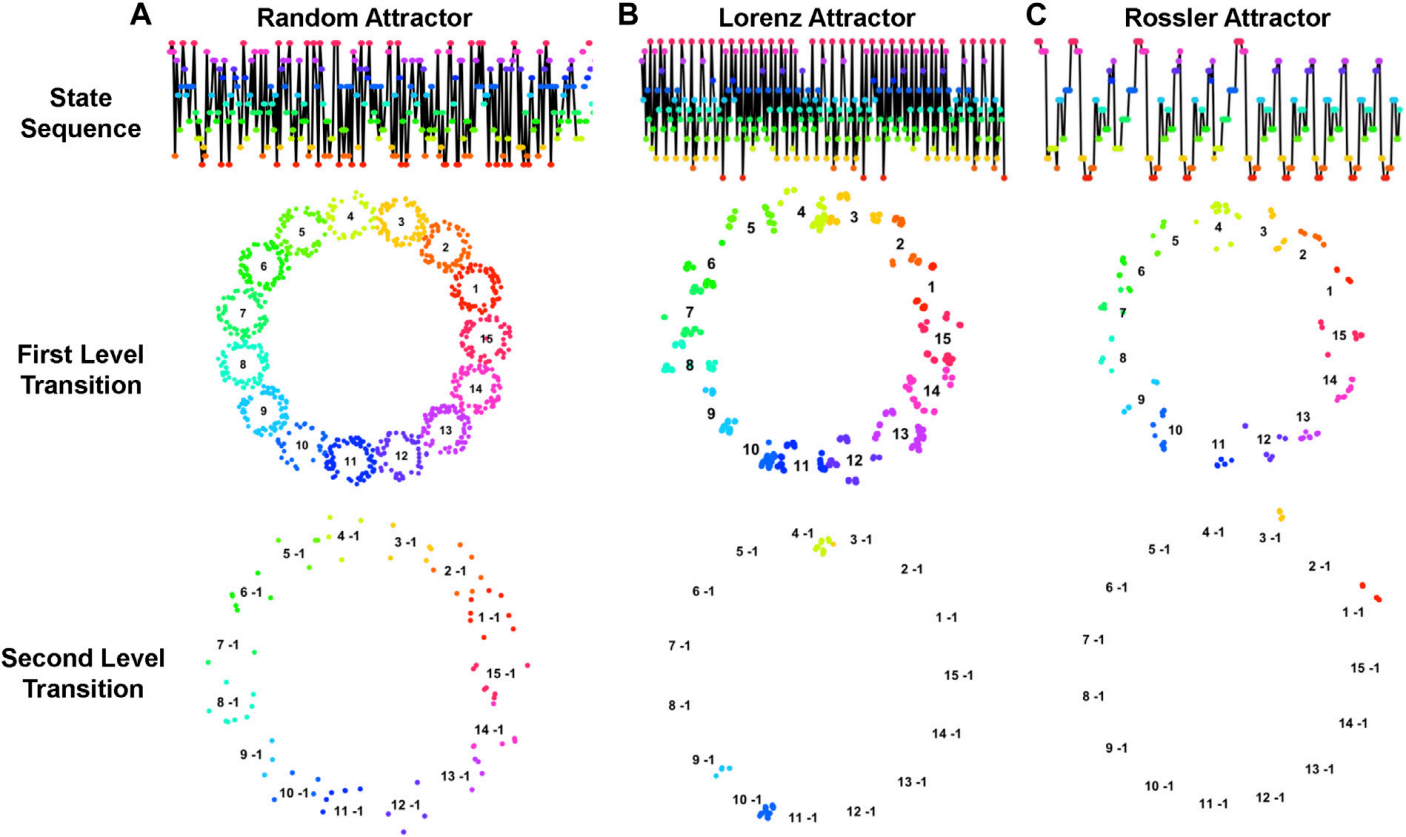
Trajectories of Dynamic Systems with Corresponding Fractal Structures. This figure illustrates the trajectories and fractal patterns of three dynamic systems: **(A)** random attractor, **(B)** Lorenz attractor, and **(C)** Rossler attractor. The top layer figures indicate the trajectories of the systems, the second- and third-layer figures illustrate the corresponding fractal structures of first- and second-level transitions. The topological structures of fractals characterize the dynamic properties of the systems.

Notably, different trajectory patterns form various fractal structures that reveal diverse dynamic characteristics of the corresponding systems. As demonstrated in Figure 6, trajectories of three different dynamic systems, including random, Lorenz, and Rossler attractors, along with their corresponding fractal structures in the first- and second-level transitions are quite different. It is noteworthy that systems with more randomness typically yield a less informative fractal structure, whereas systems with specific patterns yield a more distinctive fractal structure that is characteristically unique. Thus, analyzing the topological structure of multilevel fractals increases the resolution of dynamic system properties.

**FIGURE 7:**
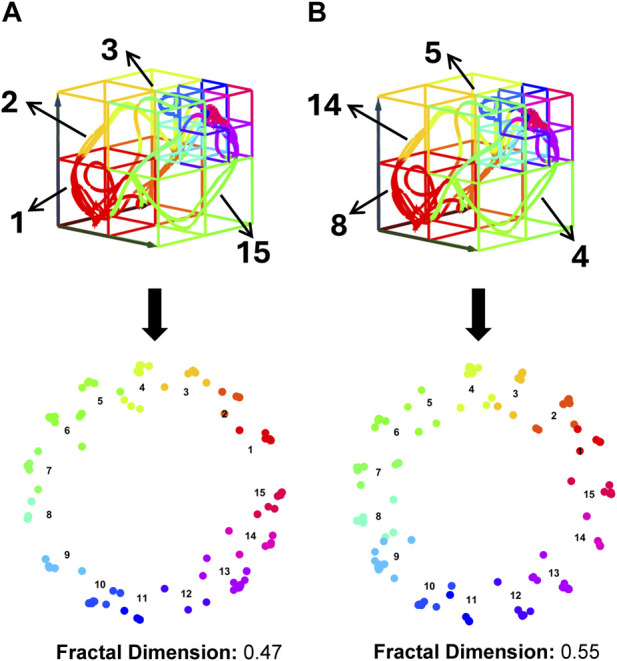
Impact of Subspace Label Assignment on Dynamic Feature Characterization. Both panels **(A, B)** display identical trajectories within the same heterogeneous state-space structure, yet they have different subspace label assignments. These differences lead to the distinct fractal structures shown in the lower layers of each panel, with varying fractal dimension values. Fractal dimension is used here to quantify the complexity of fractal structures, where higher values indicate increased complexity and greater detail retention across scales.

However, fractal representation is sensitive to the categorical labels, which are presented as a sequence of consecutive positive integers from 1 to K , each indicating a specific subspace within the state-space. As Figure 7 illustrates, even when the same trajectory underlies the same heterogeneous state-space structure, various fractal structures can emerge due to different subspace label assignments. This variability significantly influences the effectiveness of dynamic characterization. Therefore, since the dynamic characteristics of the system are derived by analyzing the fractal topology and complexity, optimizing subspace label assignments is crucial for achieving the most accurate fractal representation.

However, determining the optimal subspace label assignment is a challenging task. For example, to evaluate all possible 45 subspace assignments would be 45! (approximately 1.1962e+56) scenarios, making it impractical to exhaustively test all permutations to find the best assignment. To address this challenge, we propose a novel Genetic Algorithm (GA) to achieve a heuristic solution for optimizing subspace label assignment, as illustrated in Algorithm 1. GA is a type of evolutionary algorithm which generates solutions to problems inspired by natural selection (Holland, 1992).

Algorithm 1 Genetic Algorithm for Optimizing Label Arrangements.

 **INPUT:**
  
S : Initial sequence from which to create the trajectory
  
K : Number of distinct labels (derived from S if not provided)
  
l.size : Size of the sample Pool
  
slt : Number of instances to select for reproduction
  
pm : Mutation probability
  
ga.iter : Number of iterations for the genetic algorithm
1 **BEGIN:**
2  *//Initialize GA parameters*
3 $GAobj=GNW\left(S,K\right)$ *//Create network structure representing the trajectory of* S

4  *//Generate initial population*
5 $SamplePool=GAinit\left(K,l.size\right)$ *//Create a sample pool of sequence for GA*
6  *//Genetic algorithm main loop*
7  **FOR** iter=1 **TO** ga.iter **DO**
8   *//Evaluate fractal dimension of each instance in the sample pool*
9   **FOR EACH** instance **IN** SamplePool **DO**
10 $fitness\left[instance\right]=Fitness\left(instance,GAobj\right)$

11   **END FOR**
12   *//Select top individuals for reproduction*
13 $Selected=select\_top\left(fitness,slt\right)$

14   *//Update sampling pool through reproduction and mutation*
15 $SamplePool=reproduce\_and\_mutate\left(Selected,pm\right)$

16   *//Optional: Convergence check to break loop early*
17   **IF** $check\_convergence\left(fitness\right)$ **DO**
18    **BREAK**
19   **END IF**
20  **END FOR**
21  *//Determine the best solution*
22 $BestSolution=find\_best\left(SamplePool\right)$

23  **RETURN** BestSolution

*Fitness function returns the fractal dimension of the fractal structure generated by the input instance.

In this study, we modified the GA as follows:

• Initial Population: Started with 50,000 random subspace label assignments, each offering a unique labeling approach within the EEG state-space.
• Evaluation: Each assignment is assessed for fractal complexity to gauge effectiveness in describing the underlying trajectory structure.
• Selection and Generation: Post-evaluation, another 50,000 assignments are generated using genetic crossover and mutation techniques to explore new solutions.
• Optimization: Assignments with the highest fractal complexity, indicative of effective system dynamics capture, are selected.
• Iteration: This cycle of generation, evaluation, and optimization continues until a fractal complexity threshold is reached or no further improvements are observed.

Note that fractal complexity in this study is measured using the Minkowski fractal dimension, which involves covering the fractal with boxes of a specific size and counting the number needed to completely cover the fractal. This process is repeated with progressively smaller boxes (Hunt et al., 1939). The Minkowski fractal dimension for a fractal F can be mathematically expressed as Eq. 5:

$$\dim_{box}\left(F\right)=\lim_{\varepsilon \to 0}\frac{\log\xi \left(\varepsilon \right)}{\log\frac{1}{\varepsilon }}$$ (5)

where ξ denotes the number of boxes with a side length of ε . A higher Minkowski dimension suggests a more complex fractal, implying that it retains richer information.

#### 4.2.3 Generalized heterogeneous recurrence quantification analysis

The fractal representation clusters the system’s trajectory at multiple scales with fractal structures, which demonstrate the heterogeneous recurrence dynamics of a system on the two-dimensional coordinates. To effectively capture this heterogeneity in system recurrences, a new measurement approach has been developed that employs the fractal structure for quantifying these heterogeneous recurrences (Yang and Chen, 2014; Chen and Yang, 2015; Chen and Yang, 2016). Rather than treating all recurrences uniformly, this method, known as HRQA, specifically characterizes recurrent patterns based on the diverse states or transitions that are mapped onto the fractal structure, thereby enhancing the analytical capabilities of recurrence quantifiers. Chen and Yang derived a series of HRQA methodologies based on this fractal representation (Yang et al., 2020). However, traditional HRQA methods encounter scalability issues when attempting to quantify transitions at different levels. In response to this challenge, this research introduces a generalized HRQA system that addresses scalability issues to assess system recurrences. This advanced system allows for a more nuanced analysis of the dynamics inherent within different level transitions.

To quantify the fractal representation, the first step is to identify the sets of states falling into different level transitions in the fractal representation. Since the IFS assigns unique addresses in the circles to clusters of state sets, we define these heterogeneous recurrence sets $C_{k_{1},k_{2},..,k_{N}}$ as Eq. 6:

$$C_{k_{1},k_{2},..,k_{N}}=\left\{f\left(k_{1}|k_{2},\ldots ,k_{N}\right):L\left(s_{t}\right)=k_{1},L\left(s_{t-1}\right)=k_{2},\ldots ,L\left(s_{t-N+1}\right)=k_{N},\forall k_{t}\in K\right\}$$ (6)

Here, the subscript $k_{1},k_{2},..,k_{N}$ represents an $N^{th}$ -level transition sequence. For instance, $C_{k_{1}}=\left\{L\left(s_{t}\right)=k_{1}\right\}$ represents the recurrence set of first-level transition, and $C_{k_{1},k_{2}}=\left\{L\left(s_{t}\right)=k_{1},L\left(s_{t-1}\right)=k_{2}\right\}$ represents the recurrence set of the second-level transition. Notably, we also define $C_{\phi }$ as zero-level transition to represent overall transitions without specifying any transition pattern. This allows for the investigation and quantification of the system dynamics from a comprehensive system perspective. To simplify, we will use N to indicate the $k_{1},k_{2},..,k_{N}$ in the subsequent discussion. The generalized HRQA metrics are depicted in the following section.

##### 4.2.3.1 Heterogeneous recurrence rate (HRR)

$$HRR\left(N\right)={\left(\overset{═}{C}/L\right)}^{2}$$ (7)

HRR quantifies the proportion of a specific $N^{th}$ -level transition N occurred in an observed sequence. Note that $\overset{═}{C}$ represents the cardinality of $C_{k_{1},k_{2}\ldots ,k_{N}}$ and L indicates the length of the observed sequence.

##### 4.2.3.2 Heterogeneous recurrence mean (Hmean)

To scale the HRQA for different $N^{th}$ -level transition, we define an adjusted distance $d_{i,j}^{N}$ for two addresses i and j for each $C_{k_{1},k_{2}\ldots ,k_{N}}$ as $d_{i,j}^{N}=d_{i,j}/\alpha^{N}$ , where $d_{i,j}$ is the original distance, α is the scaling factor in Eq. 4, and N indicates the transition level. Then the generalized central tendency, variance tendency, skewness, and kurtosis of one local fractal cluster for $N^{th}$ -level transition are quantified as in Eqs 8–13 shown below, respectively.

$$HMean\left(N\right)=\frac{\sum_{i=1}^{\overset{═}{C}}\sum_{j=i+1}^{\overset{═}{C}}d_{i,j}^{N}}{\overset{═}{C}\left(\overset{═}{C}-1\right)/2}$$ (8)

##### 4.2.3.3 Heterogeneous recurrence variance (HVar)

$$HVar\left(N\right)=\sum_{i=1}^{\overset{═}{C}}\frac{\sum_{j=i+1}^{\overset{═}{C}}{\left(d_{i,j}^{N}-HMean\left(N\right)\right)}^{2}}{\overset{═}{C}\left(\overset{═}{C}-1\right)/2}$$ (9)

##### 4.2.3.4 Heterogeneous recurrence skewness (HSkew)

$$HSkew\left(N\right)=\frac{\sum_{i=1}^{\overset{═}{C}}\frac{\sum_{j=i+1}^{\overset{═}{C}}{\left(d_{i,j}^{N}-HMean\left(N\right)\right)}^{3}}{\overset{═}{C}\left(\overset{═}{C}-1\right)/2}}{HVa{r\left(N\right)}^{\frac{3}{2}}}$$ (10)

##### 4.2.3.5 Heterogeneous recurrence kurtosis (HKurtosis)

$$HKurtosis\left(N\right)=\frac{\sum_{i=1}^{\overset{═}{C}}\frac{\sum_{j=i+1}^{\overset{═}{C}}{\left(d_{i,j}^{N}-HMean\left(N\right)\right)}^{4}}{\overset{═}{C}\left(\overset{═}{C}-1\right)/2}}{HVa{r\left(N\right)}^{2}}$$ (11)

##### 4.2.3.6 Heterogeneous recurrence entropy (HENT)

$$HENT\left(N\right)=-\sum_{b=1}^{B}\mathrm{Pr}\left(b\right)\ln\;\left(\mathrm{Pr}\;\left(b\right)\right)$$ (12)

##### 4.2.3.7 Heterogeneous recurrence gini index (HGini)

$$HGini\left(N\right)=1-\sum_{b=1}^{B}{\mathrm{Pr}\left(b\right)}^{2}$$ (13)

Note that the calculation of $HENT\left(N\right)$ utilizes Shannon entropy, based on the probability distribution derived from the distance matrix $d_{i,j}^{N}$ . The histogram of distance matrix $d^{N}$ is segmented into *B* qual bins, ranging from 0 to $\max\left(d^{N}\right)$ . Consequently, for every bin b up to B, the probability of b is defined as Eq. 14:

$$\mathrm{Pr}\left(b\right)=\frac{1}{\overset{═}{C}\left(\overset{═}{C}-1\right)}\#\left\{\frac{b-1}{B}\max\left(d^{N}\right)<d_{i,j}^{N}\leq \frac{b}{B}\max\left(d^{N}\right)\right\}$$ (14)

We deployed the proposed generalized HRQA to quantify the fractal representations derived from the embedded EEG. In this research, we addressed different resolutions of dynamic to the second-level transitions. A total $7+45\times 7+45^{2}\times 7=14497$ HRQA metrics that delineate complex dynamic brain activity were then extracted for emotion recognition.

### 4.3 Phase 3: supervised ensemble learning

The final phase of our methodology is to develop a supervised machine learning model that classifies the outcome using HRQA metrics as the input. We chose ensemble learning for its ability to handle complex, nonlinear patterns and relationships within the data while achieving high accuracy in classifying the outcome. We evaluated three decision-tree-based ensemble machine learning algorithms, the adaptive boosting method (Adaboost), random forest classification (Random Forest), and extreme gradient boosting (XGBoost), for accurately identifying the four emotions.

Decision-tree-based ensemble machine learning methods effectively handle complex nonlinear relationships by integrating multiple decision trees. These methods continuously refine the model by adding new trees specifically designed to correct errors identified in existing trees. The methods evaluated in our methodology differ primarily in their training approaches: XGboost and Adaboost use boosting to focus on correcting mispredictions by adjusting data weights, while Random Forest employs bagging, sampling equally across data points. These ensemble strategies surpass single tree models by leveraging a majority vote from various trees, thus expanding the solution space and reducing overfitting through averaged outcomes.

Although tree-based models are effective at capturing complex relationships in data, their efficiency and performance can be significantly influenced by the number of predictors. These models are particularly sensitive to the inclusion of irrelevant or noisy predictors, which can increase model complexity and lead to a higher risk of overfitting, where the model learns the noise in the training data rather than the underlying patterns (Hu and Li, 2022). To overcome this issue, we employed the Least Absolute Shrinkage and Selection Operator (LASSO) for variable selection to reduce the number of HRQA metrics used in developing our emotion recognition models.

LASSO is particularly effective for models burdened by high-dimensional data, as it helps in reducing the risk of overfitting by imposing a constraint on the sum of the absolute values of the model parameters. This regularization process not only shrinks less important feature coefficients to zero but also simplifies the model by retaining only those variables that significantly contribute to the predictive power (Roth, 2004).

We executed the LASSO algorithm 30 times and selected metrics that consistently had non-zero coefficients across these runs. Table 1 illustrates the final number of HRQA metrics selected for each emotion. Our results indicate that the emotions ‘Neutral’ and ‘Sad’ are associated with a broader range of dynamic characteristics of brain activity, while ‘Fear’ and ‘Happy’ are linked to relatively fewer features.

**TABLE 1:** Number of LASSO selected HRQA metrics for each emotion.

Emotion	Number of selected metrics
Neutral	216
Sad	270
Fear	89
Happy	108

To identify the four emotions based on their dynamic characteristics extracted from LASSO selected HRQA metrics, we tailored a classification model for each specific emotion. We evaluated three supervised ensemble learning methods, AdaBoost, XGBoost, and Random Forest, for emotion recognition. For each method we used the One-vs-All (OvA) strategy, where each emotion was classified independently as the positive class against all others grouped as the negative class. To ensure the robustness and reliability of our models, we adopted a rigorous testing protocol. The data was randomly split into a training dataset (90% of the total dataset) and a testing dataset (remaining 10% of the total dataset) to prevent any potential bias in model training. Then the training dataset was used to develop three different models (AdaBoost, XGBoost, and Random Forest) for each emotion (neutral, sad, fear, happy); this process was repeated 30 times with each model to ensure stability and consistency in the results. After training the model, the testing dataset was used to validate the performance of each model. Performance was quantitatively assessed by comparing the predicted labels against the actual labels from the testing set, calculating both the average and the standard deviation. In addition, we conducted sensitivity analyses on the emotion recognition models to investigate which dynamic characteristics are strongly associated with specific emotions. This analysis helped identify key features that significantly influence the models’ ability to accurately classify different emotional states.

We assessed the effectiveness of ensemble learning models for emotion recognition using two performance metrics: accuracy and AUC. Accuracy is defined as $\left(TP+TN\right)/\left(TP+TN+FP+FN\right)$ , where True Positives (TP) represent actual positives correctly predicted as positive, True Negatives (TN) represent actual negatives correctly predicted as negative, False Positives (FP) indicate actual negatives incorrectly predicted as positive, and False Negatives (FN) refer to actual positives incorrectly predicted as negative. The ROC curve is plotted with false positive rate (1-specificity) on the *x*-axis against the true positive rate (sensitivity) on the *y*-axis at various threshold settings. Specifically, $\text{sensitivity}=TN/\left(TN+FP\right)$ and $\text{specificity}=TP/\left(TP+FN\right).$ AUC represents the area under the ROC curve, providing a single measure of overall model performance across all classification thresholds. It is particularly valuable in the presence of biased datasets, as it evaluates the model’s ability to discriminate between classes without being influenced by class imbalance (Nahm, 2022). A higher AUC value indicates better model performance, with 1.0 representing perfect discrimination and 0.5 indicating no discriminative power beyond random chance.

To achieve optimal performance, we applied grid search combined with 10-fold cross-validation to fine-tune the hyperparameter settings for the supervised ensemble learning methods, including Adaboost, Random Forest, and XGBoost. The hyperparameters yielding the highest F1 score (calculated as 2⋅TP/(2⋅TP + FP + FN)) on the validation dataset were selected. This comprehensive tuning process involved exhaustively searching through a predefined set of hyperparameters to find the optimal combination, ensuring that each model was finely adjusted to achieve the best possible performance. For Adaboost, we created an ensemble of 500 weak learners without resampling with replacement and used the Breiman method for adjusting weights. For Random Forest, we built 800 trees, each considering 30 features at each split, and used a 0.5 threshold for classification. For XGBoost, we trained 500 deep trees to solve a binary classification problem using logistic regression.

## 5 Results

We developed a comprehensive methodology consisting of three phases to identify four emotions by analyzing the corresponding complex dynamic characteristics in EEG. In this section, we discussed the performance of the proposed methodology in three perspectives. We initially compared the performance of three ensemble learning models: AdaBoost, Random Forest, and XGBoost. Then, we discussed the performance of each individual emotion identification model under XGBoost. Finally, an overall performance comparison with other models using the same dataset was conducted.

### 5.1 Model performance of AdaBoost, random forest, and XGBoost

To evaluate which ensemble learning model had the best performance for emotion recognition, accuracy and AUC was calculated for each specific emotion then averaged for each model. Table 2 demonstrates that XGBoost and Random Forest consistently achieved high accuracy and AUC, signifying excellent stability across multiple trials, whereas AdaBoost did not. Since both Random Forest and XGBoost achieved at least 0.75 in both accuracy and AUC, this suggests that dynamic transition properties of brain activity extracted from high-dimensional EEG signals using the MHRA methodology, can effectively recognize emotions. Given that accuracy was our primary performance criterion, XGBoost with an average accuracy of 0.7885 and an AUC of 0.7552 was selected as the best model for emotion recognition.

**TABLE 2:** Performance of each ensemble model of all four emotions.

Method	Accuracy	AUC
Adaboost	0.7498 (0.0118)	0.5444 (0.0631)
Random Forest	0.7518 (0.0140)	0.7666 (0.0177)
XGBoost	0.7885 (0.0116)	0.7552 (0.0207)

*Mean (Standard Deviation).

### 5.2 Performance of XGBoost for each emotion

**FIGURE 8:**
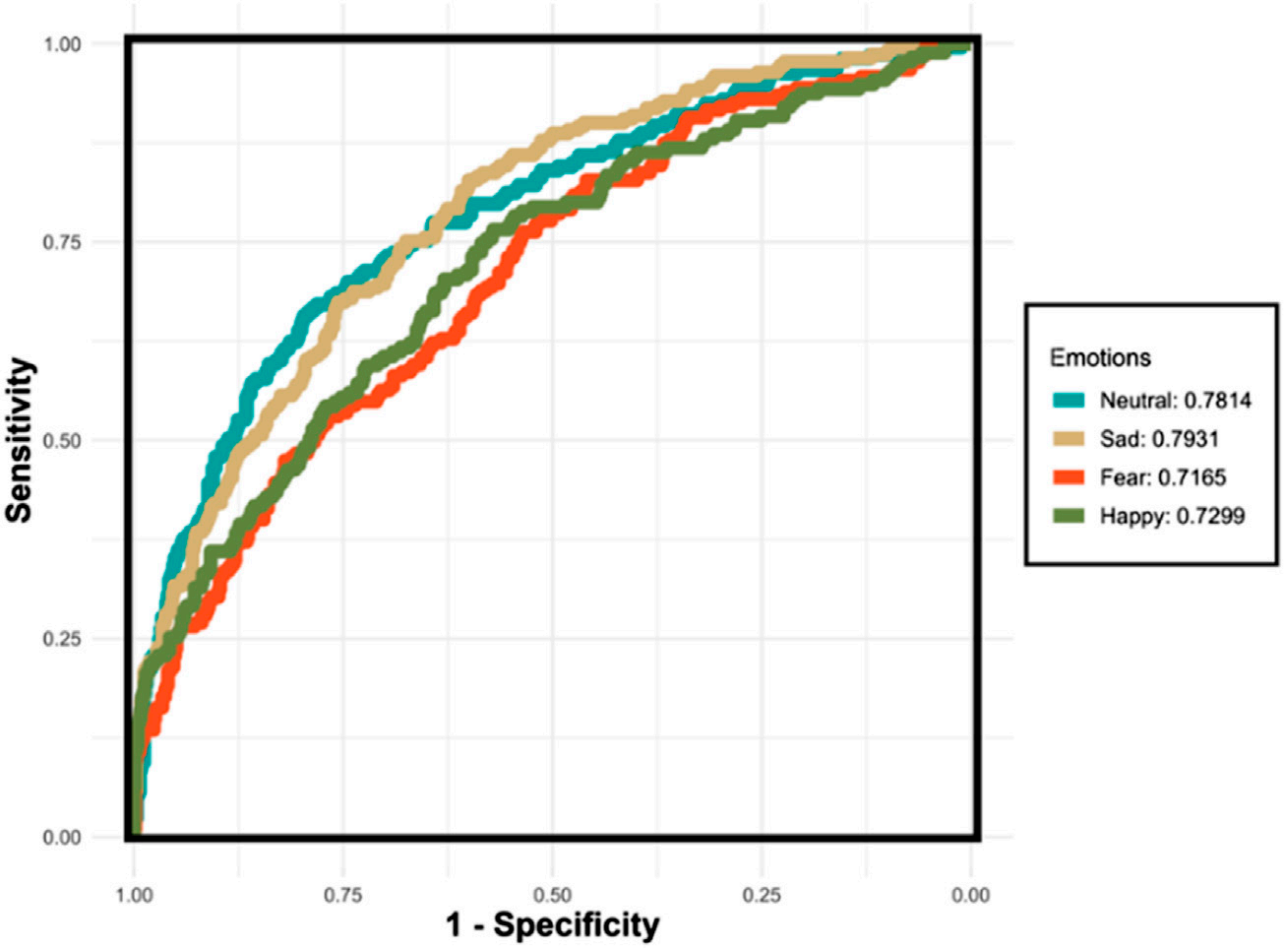
The ROC curves for the XGBoost classifier applied to the testing set using the One-vs-All (OvA) strategy for four separate emotions. The emotions “Neutral” and “Sad” exhibit relatively higher AUC values, indicating more reliable performance in distinguishing these emotions. Conversely, “Fear” and “Happy” demonstrate lower AUC values, reflecting the model’s reduced consistency in differentiating these emotions from others.

Figure 8 demonstrates the AUC curves for the XGBoost model’s performance in recognizing four distinct emotions. The curves reflect the varying levels of the model’s discriminatory ability for each emotion. The AUC for ‘Sad’ shows the highest value at 0.7931, indicating that the model is most effective at distinguishing ‘Sad’ from non-sad emotional states. ‘Neutral’ also demonstrates a robust performance with an AUC of 0.7814. However, the AUCs for ‘Fear’ and ‘Happy’ are lower, at 0.7165 and 0.7299 respectively, suggesting challenges in the model’s ability to consistently differentiate these emotions from others. The lower AUC for ‘Fear’ indicates a particular difficulty in discrimination, which could be due to the nuanced nature of fear as an emotion. Conversely, despite ‘Happy’ having the highest accuracy, its AUC indicates less consistency in distinguishing happiness, likely due to overlapping features with other emotions.

In this section, we demonstrated the performance of XGBoost into each emotion model, as shown in Table 3. The results indicate that all the emotion models can achieve at least 0.77 for accuracy and at least 0.71 for the AUC. The model excels in recognizing ‘Happy’ emotions, achieving the highest accuracy of 0.8127. The accuracies and AUCs for ‘Neutral’ and ‘Sad’ are relatively higher and more consistent, suggesting more reliable performance for these emotions. Conversely, the AUCs for ‘Fear’ and ‘Happy’ are lower and show greater variability, reflecting differences in the model’s ability to consistently distinguish these emotions from others. The small standard deviations associated with these metrics across all emotions underscore the model’s stability and reliability in performance across multiple iterations or subsets of the dataset.

**TABLE 3:** Performance of XGBoost method for each emotion.

Emotion	Proportion (%)	Accuracy	AUC
Neutral	27.09	0.7790 (0.0009)	0.7814 (0.0164)
Sad	27.27	0.7868 (0.0102)	0.7931 (0.0196)
Fear	24.49	0.7757 (0.0137)	0.7165 (0.0251)
Happy	21.15	0.8127 (0.0130)	0.7299 (0.0215)
**Average**	**25.00**	**0.7885 (0.0116)**	**0.7552 (0.0207**)

*Mean (Standard Deviation).

The bold values indicate the average performance of the four emotion models.

### 5.3 Performance comparison to other methodologies

To evaluate the performance of our methodology relative to other methodologies, Table 4 compares our performance to other methodologies using the same dataset: EmotionMeter, (Zheng et al., 2019b), BiHDM, (Li et al., 2019b), RGNN, (Zhong et al., 2019), Fractal-SNN, (Li et al., 2024), Saliency-based CNN, (Delvigne et al., 2022), MetaEmotionNet, (Ning et al., 2024), ST-SCGNN, (Pan et al., 2024), and MISNet (Gong et al., 2024). Our methodology not only outperformed all of these models in overall accuracy (0.7885) but also demonstrated the most stable performance among the repeated experiments, as indicated by the lowest standard deviation (0.0207).

**TABLE 4:** Accuracy of MHRA in emotion recognition (individual and overall) vs. other methods.

Authors	Methodology	Neutral	Sad	Fear	Happy	All (mean/s.d.)
Zheng et al. (2019)	EmotionMeter	0.7800	0.6300	0.6500	0.8000	0.7058/0.1701
Li et al. (2019)	BiHDM	0.7443	0.7273	0.5813	0.6350	0.6903/0.0866
Zhong et al. (2020)	RGNN	0.7516	0.9192	0.7185	0.7435	0.7384/0.0802
Li et al. (2023)	Fractal-SNN	-	-	-	-	0.6833/--------
Delvigne et al. (2023)	Saliency based CNN	-	-	-	-	0.7442/0.0476
Ning et al. (2024)	MetaEmotionNet	0.5393	0.6312	0.5052	0.7415	0.6120/0.0830
Pan et al. (2024)	ST-SCGNN	-	-	-	-	0.7637/0.5777
Gong et al. (2024)	MISNet	0.7071	0.8300	0.6319	0.8169	0.7460/0.0930
**Wang et al. (2024)**	**MHRA**	**0.7790**	**0.7868**	**0.7757**	**0.8127**	**0.7885/0.0207**

The bold values shows the results of this research.

Notably, our methodology provided the most consistent recognition performance across different emotions, with average accuracies ranging from 0.7757 to 0.8127. This consistency highlights the robustness and effectiveness of our approach in capturing the subtle dynamics of brain activity. In contrast, other methods showed varying strengths across specific emotions. For example, EmotionMeter is more effective in identifying ‘Happy’ and ‘Neutral’, BiHDM is more accurate in recognizing ‘Neutral’ and ‘Sad’, RGNN and MetaEmotionNet are specifically sensitive to ‘Sad’ and ‘Happy’, respectively, and MISNet performs better in ‘Sad’ and ‘Happy’. This implies that previous models struggle to grasp the nuanced activities in the brain, likely due to their inability to fully capture the complex characteristics of EEG signals. Collectively, this indicates that complex brain activity can be effectively characterized using dynamic recurrence properties with our novel MHRA methodology.

These results highlight the robustness and effectiveness of our approach in handling the complex, nonlinear, and nonstationary characteristics of EEG signals. Our methodology’s ability to maintain high accuracy across all emotions and its stable performance in repeated experiments underscore its reliability and potential for real-world applications. By comparing our findings with the relevant literature, it is evident that MHRA not only advances the state of the art in emotion recognition but also provides a versatile method for analyzing complex brain dynamics. This comprehensive analysis reinforces the value of our contributions to the field and demonstrates the superiority of our approach over existing methods.

**FIGURE 9:**
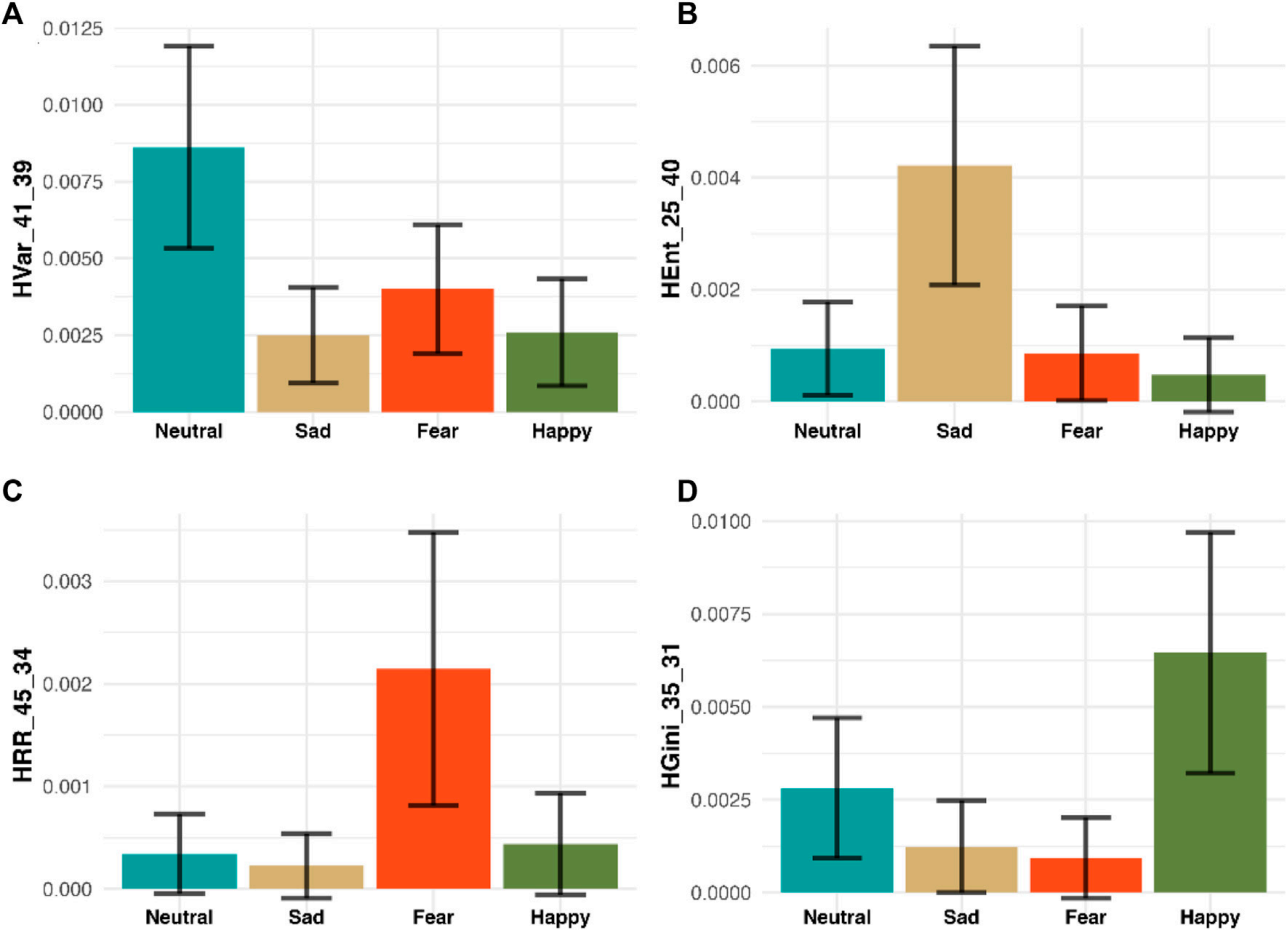
Sensitivity Analysis of Four selected HRQA Metrics. The four panels **(A–D)** display four selected HRA metrics for four emotions, respectively. Specifically, Panel A shows HVar_41_39 has the highest value in “Neutral,” Panel B demonstrates HEnt_25_40 has the highest value in “Sad,” Panel C illustrates HRR_45_34 has the highest value in “Fear,” and Panel D presents HGini_35_31 has the highest value in “Happy.” Each panel highlights a metric where one emotion scores significantly higher on average than the others, demonstrating the metric’s potential to distinctly identify that emotion from the rest.

In addition to achieving the highest accuracy in emotion recognition, our methodology offers profound insights into the specific dynamic features that drive emotional responses, thereby enhancing our understanding of complex brain activity. We demonstrate that variations in the distribution of MHRA metrics are key indicators for emotion recognition, providing robust evidence of our model’s superiority over traditional ‘black box’ methods. For example, Figure 9 presents a sensitivity analysis of how specific HRQA metrics vary in value across each emotion. Specifically, each panel is one unique HRQA that corresponds to a dynamic property that characterizes a specific transition between different subspaces within the constructed heterogeneous state-space.

Panel A displays the HVar in the transition from subspace #21 to subspace #39. Here, the ‘Neutral’ emotion exhibits the highest average, suggesting significant variability during these transitions. Panel illustrates the HEnt during transitions from subspace #25 to subspace #40, with ‘Sad’ recording the highest average, indicating pronounced entropy in these transitions. Panel C depicts the HRR between subspaces #45 and #34. Here, ‘Fear’ stands out with the highest average, reflecting a notable recurrence rate. Finally, Panel D tracks inequality HGini in the transitions from subspace #35 to subspace #31, where ‘Happy’ demonstrates the highest average, highlighting significant inequality in these transitions. Each bar chart is accompanied by a 95% confidence interval, providing a clear visual representation of how distinct MHRA metrics correlate with each emotional state.

These findings not only confirm the efficacy of our model in identifying and interpreting emotions but also provide a methodology for investigating the subtle spatiotemporal dynamics underlying brain activity related to various emotions. By analyzing these HRQA metrics, we may infer the neural mechanisms involved in emotion recognition. For instance, the high value of entropy (HEnt), referring to a high level of uncertainty, in ‘Sad’ could signify chaotic neural activity patterns associated with emotional distress or cognitive load. The high value of recurrence rate (HRR), referring to a high tendency to revisit similar patterns, in ‘Fear’ suggests a specific pattern of repetitive neural activations, possibly related to the brain’s heightened state of alertness and threat detection.

By correlating these dynamic features with known neural processes, our approach offers deeper insights into how different emotional states manifest in the brain’s activity. This enhanced understanding can contribute to developing more effective interventions and therapeutic strategies for emotional and mental health disorders. Thus, our methodology not only advances the field of emotion recognition but also provides a valuable tool for exploring the neural underpinnings of emotions.

## 6 Discussion

Understanding how emotions are processed and represented in the brain enhances our basic scientific knowledge of neurological functions. By studying EEG patterns associated with different emotions, researchers can uncover the underlying neural mechanisms that govern emotional responses and how these might differ among individuals or across different contexts. However, the complex, nonlinear, and nonstationary characteristics of EEG signals pose significant challenges for many traditional methods in this field. Numerous studies on EEG-based emotion recognition rely on deep learning techniques, as these state-of-the-art neural network-based methods are adept at detecting subtle patterns within complex EEG signals (Jafari et al., 2023). Nonetheless, the lack of transparency in deep learning algorithms represents a substantial barrier, as physicians tend to be cautious by nature, and patients are hesitant to entrust their health to a ‘black box’ algorithm. In this study, we introduced a three-phase methodology, including manifold embedding, MHRA, and supervised ensemble learning, designed to address these concerns by characterizing the dynamic features of brain activity for emotion recognition while also preserving a degree of explainability.

We employed the proposed MHRA methodology to the SJTU-SEED IV database, in Phase 1, we utilized UMAP for data embedding to address the challenge of high dimensional data. The 62-lead EEG signals were transformed into four-dimensional embedded signals that retain dynamic spatiotemporal characteristics but significantly reduced computational demands to a manageable level for further analyses. In Phase 2, the embedded EEG data underwent our novel MHRA to capture the recurrence dynamics of brain activity at high resolution. This approach not only provides a more nuanced understanding of the complex nonlinear and nonstationary EEG patterns, but also extracts robust dynamic features for emotion recognition. Importantly, our generalized HRQA metrics systematically quantify recurrences across different transition levels, offering a scalable framework for analyzing dynamic EEG properties. Finally, in Phase 3 we employed advanced ensemble learning methods and demonstrated their effectiveness in classifying emotions using LASSO selected HRQA metrics. The superior performance of our models, especially XGBoost, suggests that dynamic transition characteristics are powerful predictors for emotion recognition. Our models achieved accuracy and AUC values of 0.7885 and 0.7552, respectively, both outperforming previous studies using the same dataset. Additionally, our sensitivity analysis identified specific HRQA metrics strongly associated with each emotion, providing valuable insights into the neural dynamics underlying emotional processing that cannot be obtained using “black box” algorithms alone.

The major contribution of this research is the development of MHRA, a novel technique leveraging the recurrence theorem to characterize dynamic brain activity across multiple granularities. Unlike traditional methods, MHRA captures the complex, nonlinear, and nonstationary properties of EEG signals, providing a detailed framework for analyzing intricate brain activity patterns. By utilizing HRQA metrics, MHRA offers an interpretable analysis of EEG data, aiding researchers in understanding the neural mechanisms of emotions. This transparency is crucial for building trust and facilitating the adoption of our methodology in clinical and research settings. The insights from our MHRA approach have significant implications for advancing studies in cognitive neuroscience, affective computing, neurofeedback therapy, human-computer interaction, and educational neuroscience. Traditional approaches often struggle with the nonlinear and nonstationary nature of EEG signals, while deep learning models lack explainability. Our methodology overcomes these challenges, offering both high performance and interpretability, thus advancing the field of emotion recognition and providing an effective solution for analyzing complex brain dynamics. Our methodology offers several key advantages. First, it effectively addresses the limitations of traditional linear methods by analyzing complex nonlinear nonstationary EEG signals. Second, MHRA offers interpretability by using HRQA metrics to explain features of complex systems. This transparency is crucial for building trust and facilitating adoption in clinical settings. Third, the tree-based ensemble learning methods not only achieve high accuracy to recognize emotions but also exhibit robustness in capturing nonlinear relationships of dynamic properties.

Despite these strengths, our study has some limitations that will be explored in future research. The SJTU-SEED IV database, while comprehensive, does not fully capture the diversity or unique emotional experiences across different populations. Investigating the generalizability of our methodology to other EEG datasets and real-world scenarios is an important next step. Additionally, integrating our approach with other modalities, such as facial expressions or other physiological signals such as eye movements, could further enhance the accuracy and robustness of emotion recognition. Furthermore, our research can facilitate a deeper understanding and characterization of brain activities, with potential applications in pediatric sleep studies, the development of objective metrics for PTSD, and non-invasive early detection of neurodegenerative diseases. Future research could benefit from incorporating more advanced techniques to retain the spatiotemporal characteristics of 62-lead EEG signals, such as integrating attention mechanisms with MHRA to provide more effective characterization of neural dynamics. By pursuing these directions, we aim to refine the existing methodology and broaden its applicability, thus advancing the field of emotion recognition and its practical applications in neuroscience and healthcare.

In conclusion, this study presents a novel three-phase methodology that includes manifold embedding, MHRA, and ensemble learning for EEG-based emotion recognition. Our approach not only achieves high performance but also offers interpretable insights into the dynamic properties underlying four emotions. This methodology has significant impact on the field to advance our ability to analyze nonlinear nonstationary, dynamic data of complex systems with potential applications in healthcare, human-computer interaction, and beyond.

## Data Availability

The data analyzed in this study was obtained from the Shanghai Jiao Tong University (SJTU) Emotion EEG Dataset for Four Emotions (SEED-IV), which is a specific subset of the broader SJTU Emotion EEG Dataset (available at https://bcmi.sjtu.edu.cn/∼seed/). The following licenses/restrictions apply: the dataset is restricted to academic research use only and cannot be used for any commercial purposes. Distribution of the dataset or portions thereof is prohibited, except for small portions used to clarify academic publications or presentations. Access to the dataset is granted only after filling out, signing, and uploading the license agreement to the SEED website, followed by a review of the application. No warranty is provided with the dataset, and users must cite the relevant publications when using the dataset in their research. Requests to access these datasets should be directed to SEED Dataset (https://bcmi.sjtu.edu.cn/home/seed/index.html).

## References

[B1] Akhand M. A. H. Maria M. A. Kamal M. A. S. Murase K. (2023). Improved EEG-based emotion recognition through information enhancement in connectivity feature map. Sci. Rep. 13 (13), 13804–13817. 10.1038/s41598-023-40786-2 37612354 PMC10447430

[B2] Amiri A. Samet H. Ghanbari T. (2022). Recurrence plots based method for detecting series arc faults in photovoltaic systems. IEEE Trans. Industrial Electron. 69, 6308–6315. 10.1109/tie.2021.3095819

[B3] Asghar Q. Jalil A. Zaman M. (2020). Self-organization analysed in architecture using Voronoi tessellation and particle systems. Tech. J. 25, 1–10.

[B4] Avdan G. Chen C. Onal S. (2024). An alternative EMG normalization method: heterogeneous recurrence quantification analysis of isometric maximum voluntary contraction movements. Biomed. Signal Process Control 93, 106219. 10.1016/j.bspc.2024.106219

[B5] Avdan G. Chen C. B. Onal S. (2023). “Investigation of an alternative EMG normalization technique: recurrence quantification analysis of maximum voluntary contractions,” in IISE annual conference and expo 2023 (IISE). 10.21872/2023IISE_1909

[B6] Bazgir O. Mohammadi Z. Habibi S. A. H. (2018). “Emotion recognition with machine learning using EEG signals,” in 2018 25th Iranian conference on biomedical engineering and 2018 3rd international Iranian conference on biomedical engineering (ICBME), 1–5. IEEE.

[B7] Bouabdelli S. Meddi M. Zeroual A. Alkama R. (2020). Hydrological drought risk recurrence under climate change in the karst area of Northwestern Algeria. J. Water Clim. Change 11, 164–188. 10.2166/wcc.2020.207

[B8] Chai X. Wang Q. Zhao Y.-P. Liu X. Liu D. Bai O. (2018). Multi-subject subspace alignment for non-stationary EEG-based emotion recognition. Technol. Health Care 26, 327–335. 10.3233/thc-174739 29758967 PMC6004980

[B9] Chang H. Zong Y. Zheng W. Tang C. Zhu J. Li X. (2022). Depression assessment method: an EEG emotion recognition framework based on spatiotemporal neural network. Front. Psychiatry 12, 837149. 10.3389/fpsyt.2021.837149 35368726 PMC8967371

[B10] Chen C. Ji Z. Sun Y. Bezerianos A. Thakor N. Wang H. (2023b). self-attentive channelchannel-connectivity capsule network for EEG-based driving fatigue detection. IEEE Trans. Neural Syst. Rehabilitation Eng. 31, 3152–3162. 10.1109/TNSRE.2023.3299156 37494165

[B11] Chen C. Li Z. Wan F. Xu L. Bezerianos A. Wang H. (2022a). Fusing frequency-domain features and brain connectivity features for cross-subject emotion recognition. IEEE Trans. Instrum. Meas. 71, 1–15. 10.1109/TIM.2022.3168927

[B12] Chen C. Vong C. M. Wang S. Wang H. Pang M. (2022b). Easy Domain Adaptation for cross-subject multi-view emotion recognition. Knowl. Based Syst. 239, 107982. 10.1016/j.knosys.2021.107982

[B13] Chen C.-B. (2019). Recurrence analysis of high-dimensional complex systems with applications in healthcare and manufacturing.

[B14] Chen C. B. Wang Y. Fu X. Yang H. (2023c). Recurrence network analysis of histopathological images for the detection of invasive ductal carcinoma in breast cancer. IEEE/ACM Trans. Comput. Biol. Bioinform 20, 3234–3244. 10.1109/TCBB.2023.3282798 37276118

[B15] Chen C. B. Yang H. Kumara S. (2018). Recurrence network modeling and analysis of spatial data. Chaos 28, 085714. 10.1063/1.5024917 30180605

[B16] Chen C.-B. Yang H. Kumara S. (2019d). A novel pattern-frequency tree for multisensor signal fusion and transition analysis of nonlinear dynamics. IEEE Sens. Lett. 3, 1–4. 10.1109/lsens.2018.2884241

[B17] Chen C.-B. Yang H. Kumara S. (2017). “A novel pattern-frequency tree approach for transition analysis and anomaly detection in nonlinear and nonstationary systems,” in IIE annual conference. Proceedings, 1264–1269.

[B18] Chen D. Huang H. Bao X. Pan J. Li Y. (2023a). An EEG-based attention recognition method: fusion of time domain, frequency domain, and non-linear dynamics features. Front. Neurosci. 17, 1194554. 10.3389/fnins.2023.1194554 37502681 PMC10368951

[B19] Chen D. W. Miao R. Yang W. Q. Liang Y. Chen H. H. Huang L. (2019b). A feature extraction method based on differential entropy and linear discriminant analysis for emotion recognition. Sensors 19, 1631. 10.3390/s19071631 30959760 PMC6479375

[B20] Chen J. zhang peize Mao Z. Huang Y. Jiang D. Zhang Y. N. (2019a). Accurate EEG-based emotion recognition on combined features using deep convolutional neural networks. Ieee Access 7, 44317–44328. 10.1109/access.2019.2908285

[B21] Chen R. Imani F. Yang H. (2020). Heterogeneous recurrence analysis of disease-altered spatiotemporal patterns in multi-channel cardiac signals. IEEE J. Biomed. Health Inf. 24, 1619–1631. 10.1109/JBHI.2019.2952285 31715575

[B22] Chen R. Rao P. Lu Y. Reutzel E. Yang H. (2019c). Recurrence network analysis of design-quality interactions in additive manufacturing. Sci. Total Environ., 135907. 10.1016/j.addma.2021.101861 35527803 PMC9074762

[B23] Chen Y. Yang H. (2015). “Heterogeneous recurrence T-squared charts for monitoring and control of nonlinear dynamic processes,” in 2015 IEEE international conference on automation science and engineering (CASE), 1066–1071.

[B24] Chen Y. Yang H. (2016). Heterogeneous recurrence representation and quantification of dynamic transitions in continuous nonlinear processes. Eur. Phys. J. B 89, 155. 10.1140/epjb/e2016-60850-y

[B25] Cheng C. Kan C. Yang H. (2016). Heterogeneous recurrence analysis of heartbeat dynamics for the identification of sleep apnea events. Comput. Biol. Med. 75, 10–18. 10.1016/j.compbiomed.2016.05.006 27228436

[B26] Dan Y. Tao J. Fu J. Zhou D. (2021). Possibilistic clustering-promoting semi-supervised learning for EEG-based emotion recognition. Front. Neurosci. 15, 690044. 10.3389/fnins.2021.690044 34276295 PMC8281971

[B27] Delvigne V. Facchini A. Wannous H. Dutoit T. Ris L. Vandeborre J.-P. (2022). A saliency based feature fusion model for EEG emotion estimation, 3170–3174. 10.1109/EMBC48229.2022.9871720 36086672

[B28] Donner R. V. Small M. Donges J. F. Marwan N. Zou Y. Xiang R. (2011). Recurrence-based time series analysis by means of complex network methods. Int. J. Bifurcation Chaos 21, 1019–1046. 10.1142/s0218127411029021

[B29] Donner R. V. Zou Y. Donges J. F. Marwan N. Kurths J. (2010). Recurrence networks-a novel paradigm for nonlinear time series analysis. New J. Phys. 12, 033025. 10.1088/1367-2630/12/3/033025

[B31] Eckmann J.-P. Oliffson Kamphorst S. Ruelle D. (1987). Recurrence plots of dynamical systems. Europhys. Lett. (EPL) 4, 973–977. 10.1209/0295-5075/4/9/004

[B32] Elgamal T. Hefeeda M. (2015). Analysis of PCA algorithms in distributed environments. Available at: https://arxiv.org/abs/1503.05214v2 (Accessed April 23, 2024).

[B33] Eroglu D. Marwan N. Prasad S. Kurths J. (2014). Finding recurrence networks’ threshold adaptively for a specific time series. Nonlinear Process Geophys 21, 1085–1092. 10.5194/npg-21-1085-2014

[B34] Gao Z. Cui X. Wan W. Gu Z. (2019). Recognition of emotional states using multiscale information analysis of high frequency EEG oscillations. Entropy 21, 609. 10.3390/E21060609 33267323 PMC7515095

[B35] Gong M. Zhong W. Ye L. Zhang Q. (2024). MISNet: multi-source information-shared EEG emotion recognition network with two-stream structure. Front. Neurosci. 18, 1293962. 10.3389/fnins.2024.1293962 38419660 PMC10899343

[B36] Hatami N. Gavet Y. Debayle J. (2019). Bag of recurrence patterns representation for time-series classification. Pattern Analysis Appl. 22, 877–887. 10.1007/s10044-018-0703-6

[B37] Haynes J. D. Rees G. (2006). Decoding mental states from brain activity in humans. Nat. Rev. Neurosci. 7, 523–534. 10.1038/nrn1931 16791142

[B38] Holland J. H. (1992). Genetic algorithms. Sci. Am. 267, 66–72. 10.1038/scientificamerican0792-66 1411454

[B39] Houssein E. H. Hammad A. Ali A. A. (2022). Human emotion recognition from EEG-based brain–computer interface using machine learning: a comprehensive review. Neural Comput. Appl. 34 (34), 12527–12557. 10.1007/s00521-022-07292-4

[B40] Hu L. Li L. (2022). Using tree-based machine learning for health studies: literature review and case series. Int. J. Environ. Res. Public Health 19, 16080. 10.3390/ijerph192316080 36498153 PMC9736500

[B41] Hunt F. V. Beranek L. L. Maa D. Y. (1939). Analysis of sound decay in rectangular rooms. J. Acoust. Soc. Am. 11, 80–94. 10.1121/1.1916010

[B42] Jafari M. Shoeibi A. Khodatars M. Bagherzadeh S. Shalbaf A. García D. L. (2023). Emotion recognition in EEG signals using deep learning methods: a review. Comput. Biol. Med. 165, 107450. 10.1016/j.compbiomed.2023.107450 37708717

[B43] Jellinger K. A. (2003). Functional magnetic resonance imaging: an introduction to methods. Eur. J. Neurol. 10, 751–752. 10.1046/j.1468-1331.2003.00657.x

[B44] Kan C. Cheng C. Yang H. (2016). Heterogeneous recurrence monitoring of dynamic transients in ultraprecision machining processes. J. Manuf. Syst. 41, 178–187. 10.1016/j.jmsy.2016.08.007

[B45] Khoo M. C. K. Webber C. L. Zbilut J. P. (1996). Assessing deterministic structures in physiological systems using recurrence plot strategies. Bioeng. approaches Pulm. physiology Med., 137–148. 10.1007/978-0-585-34964-0_8

[B46] Li C. Chen B. Zhao Z. Cummins N. Schuller B. W. (2021b). Hierarchical attention-based temporal convolutional networks for eeg-based emotion recognition. 10.1109/icassp39728.2021.9413635

[B47] Li J. Li S. Pan J. Wang F. (2021a). Cross-subject EEG emotion recognition with self-organized graph neural network. Front. Neurosci. 15, 611653. 10.3389/fnins.2021.611653 34177441 PMC8221183

[B48] Li W. Fang C. Zhu Z. Chen C. Song A. (2024). Fractal spiking neural network scheme for EEG-based emotion recognition. IEEE J. Transl. Eng. Health Med. 12, 106–118. 10.1109/JTEHM.2023.3320132 38088998 PMC10712674

[B49] Li Y. Zheng W. Cui Z. Zong Y. Ge S. (2019a). EEG emotion recognition based on graph regularized sparse linear regression. Neural Process Lett. 49, 555–571. 10.1007/s11063-018-9829-1

[B50] Li Y. Zheng W. Wang L. Zong Y. Qi L. Cui Z. (2019b). A novel Bi-hemispheric discrepancy model for EEG emotion recognition. Available at: http://arxiv.org/abs/1906.01704.

[B51] Lindquist K. A. Wager T. D. Kober H. Bliss‐Moreau E. Barrett L. F. (2012). The brain basis of emotion: a meta-analytic review. Behav. Brain Sci. 35, 121–143. 10.1017/s0140525x11000446 22617651 PMC4329228

[B52] Liu H. Zhang Y. Li Y. Kong X. (2021). Review on emotion recognition based on electroencephalography. Front. Comput. Neurosci. 15, 758212–758215. 10.3389/fncom.2021.758212 34658828 PMC8518715

[B53] Liu X. Li T. Tang C. Xu T. Chen P. Bezerianos A. (2019). Emotion recognition and dynamic functional connectivity analysis based on EEG. IEEE Access 7, 143293–143302. 10.1109/access.2019.2945059

[B54] Liu Y. Sourina O. Nguyen M. K. (2010). “Real-time EEG-based human emotion recognition and visualization,” in Proceedings - 2010 international conference on cyberworlds, CW 2010, 262–269.

[B55] Lucarini V. Faranda D. de Freitas J. M. M. Holland M. Kuna T. Nicol M. (2016). Extremes and recurrence in dynamical systems. John Wiley \and Sons.

[B56] Marwan N. (2008). A historical review of recurrence plots. Eur. Phys. J. Special Top. 164, 3–12. 10.1140/epjst/e2008-00829-1

[B57] Marwan N. Carmen R. M. Thiel M. Kurths J. (2007a). Recurrence plots for the analysis of complex systems. Phys. Rep. 438, 237–329. 10.1016/j.physrep.2006.11.001

[B58] Marwan N. Kurths J. Saparin P. (2007b). Generalised recurrence plot analysis for spatial data. Phys. Lett. Sect. A General, Atomic Solid State Phys. 360, 545–551. 10.1016/j.physleta.2006.08.058

[B59] Mcinnes L. Healy J. Melville J. (2020). UMAP: uniform manifold approximation and projection for dimension reduction.

[B60] Meilă M. Zhang H. (2024). Manifold learning: what, how, and why. Annu. Rev. Stat. Appl. 11, 393–417. 10.1146/annurev-statistics-040522-115238

[B61] Mosavi A. Faghan Y. Ghamisi P. Duan P. Ardabili S. F. Salwana E. (2020). Comprehensive review of deep reinforcement learning methods and applications in economics. Mathematics 8, 1640. 10.3390/MATH8101640

[B62] Murugappan M. Murugappan S. (2013) “Human emotion recognition through short time Electroencephalogram (EEG) signals using Fast Fourier Transform (FFT),” in Proceedings - 2013 IEEE 9th international colloquium on signal processing and its applications. IEEE, 289–294. 10.1109/CSPA.2013.6530058

[B63] Nahm F. S. (2022). Receiver operating characteristic curve: overview and practical use for clinicians. Korean J. Anesthesiol. 75, 25–36. 10.4097/kja.21209 35124947 PMC8831439

[B64] Ning X. Wang J. Lin Y. Cai X. Chen H. Gou H. (2024). MetaEmotionNet: spatial–spectral–temporal-based attention 3-D dense network with meta-learning for EEG emotion recognition. IEEE Trans. Instrum. Meas. 73, 1–13. 10.1109/tim.2023.3338676

[B65] Pan J. Liang R. He Z. Li J. Liang Y. Zhou X. (2024). ST-SCGNN: a spatio-temporal self-constructing graph neural network for cross-subject EEG-based emotion recognition and consciousness detection. IEEE J. Biomed. Health Inf. 28, 777–788. 10.1109/JBHI.2023.3335854 38015677

[B66] Pang M. Wang H. Huang J. Vong C. M. Zeng Z. Chen C. (2024). Multi-scale masked autoencoders for cross-session emotion recognition. IEEE Trans. Neural Syst. Rehabilitation Eng. 32, 1637–1646. 10.1109/TNSRE.2024.3389037 38619940

[B67] Peng B. Chen C.-B. (2023). “Multiscale dynamic transition analysis of solar radiation prediction,” in IISE annual conference and expo 2023. 10.21872/2023IISE_1787

[B68] Peng Y. Liu H. Li J. Huang J. Lu B.-L. Kong W. (2023). Cross-session emotion recognition by joint label-common and label-specific EEG features exploration. Ieee Trans. Neural Syst. Rehabilitation Eng. 31, 759–768. 10.1109/tnsre.2022.3233109 37015629

[B69] Pouyet E. Rohani N. Katsaggelos A. K. Cossairt O. Walton M. (2018). Innovative data reduction and visualization strategy for hyperspectral imaging datasets using t-SNE approach. Pure Appl. Chem. 90, 493–506. 10.1515/pac-2017-0907

[B70] Roth V. (2004). The generalized LASSO. IEEE Trans. Neural Netw. 15, 16–28. 10.1109/TNN.2003.809398 15387244

[B71] Roweis S. T. Saul L. K. (1979). Nonlinear dimensionality reduction by locally linear embedding. Science 290, 2323–2326. 10.1126/science.290.5500.2323 11125150

[B73] Shu Z. R. Chan P. W. Li Q. S. He Y. C. Yan B. W. (2021). Investigation of chaotic features of surface wind speeds using recurrence analysis. J. Wind Eng. Industrial Aerodynamics 210, 104550. 10.1016/j.jweia.2021.104550

[B74] Si X. Huang D. Sun Y. Huang S. W. He H. Ming D. (2023). Transformer-based ensemble deep learning model for EEG-based emotion recognition. Brain Sci. Adv. 9, 210–223. 10.26599/bsa.2023.9050016

[B75] Thornton M. A. Tamir D. I. (2017). Mental models accurately predict emotion transitions. Proc. Natl. Acad. Sci. U. S. A. 114, 5982–5987. 10.1073/pnas.1616056114 28533373 PMC5468631

[B76] Tian Z. Huang D. Zhou S. Zhao Z.-D. Jiang D. (2021). Personality first in emotion: a deep neural network based on electroencephalogram channel attention for cross-subject emotion recognition. R. Soc. Open Sci. 8, 201976. 10.1098/rsos.201976 34457321 PMC8371362

[B77] Tong F. Pratte M. S. (2012). Decoding patterns of human brain activity. Annu. Rev. Psychol. 63, 483–509. 10.1146/annurev-psych-120710-100412 21943172 PMC7869795

[B78] Turchetti C. Falaschetti L. (2019). A manifold learning approach to dimensionality reduction for modeling data. Inf. Sci. (N Y) 491, 16–29. 10.1016/j.ins.2019.04.005

[B79] Van Booven D. J. Chen C. Malpani S. Mirzabeigi Y. Mohammadi M. Wang Y. (2024b). Synthetic genitourinary image synthesis via generative adversarial networks: enhancing AI diagnostic precision. 10.3390/jpm14070703 PMC11278131 39063957

[B80] Van Booven D. J. Chen C.-B. Kryvenko O. Punnen S. Sandoval V. Malpani S. (2024a). Synthetic histology images for training ai models: a novel approach to improve prostate cancer diagnosis. bioRxiv, 2001–2024. 10.1101/2024.01.25.577225

[B81] Wang F. Wu S. Zhang W. Xu Z. Zhang Y. Wu C. (2020). Emotion recognition with convolutional neural network and EEG-based EFDMs. Neuropsychologia 146, 107506. 10.1016/j.neuropsychologia.2020.107506 32497532

[B82] Wang H. Xu L. Bezerianos A. Chen C. Zhang Z. (2021). Linking attention-based multiscale CNN with dynamical GCN for driving fatigue detection. IEEE Trans. Instrum. Meas. 70, 1–11. 10.1109/TIM.2020.3047502 33776080

[B83] Wang Y. Chen C.-B. (2022). “Recurrence quantification analysis for spatial data,” in IIE annual conference. Proceedings, 1–6.

[B84] Wang Z. Chen C. Li J. Wan F. Sun Y. Wang H. (2023). ST-CapsNet: linking spatial and temporal attention with capsule network for P300 detection improvement. IEEE Trans. Neural Syst. Rehabilitation Eng. 31, 991–1000. 10.1109/tnsre.2023.3237319 37021904

[B72] Webber C. L. Marwan N. (2015). Recurrence quantification analysis. Theory and best practices, 426.

[B86] Webber Jr C. L. Zbilut J. P. (2005). Recurrence quantification analysis of nonlinear dynamical systems. Tutorials Contemp. nonlinear methods Behav. Sci. 94, 26–94.

[B87] Wolpaw J. R. Birbaumer N. (2006). Brain–computer interfaces for communication and control. 10.1017/cbo9780511545061.036 12048038

[B88] Xu T. Huang J. Pei Z. Chen J. Li J. Bezerianos A. (2023b). The effect of multiple factors on working memory capacities: aging, task difficulty, and training. IEEE Trans. Biomed. Eng. 70, 1967–1978. 10.1109/TBME.2022.3232849 37015624

[B89] Xu T. Wang H. Lu G. Wan F. Deng M. Qi P. (2023a). E-key: an EEG-based biometric authentication and driving fatigue detection system. IEEE Trans. Affect Comput. 14, 864–877. 10.1109/taffc.2021.3133443

[B90] Yang H. Chen C. B. Kumara S. (2020). Heterogeneous recurrence analysis of spatial data. Chaos 30, 013119. 10.1063/1.5129959 32013465

[B92] Yang H. Chen Y. (2014). Heterogeneous recurrence monitoring and control of nonlinear stochastic processes. Chaos 24, 013138. 10.1063/1.4869306 24697400

[B94] Yang L. Zheng W. Wang L. Zong Y. Cui Z. (2022). From regional to global brain: a novel hierarchical spatial-temporal neural network model for EEG emotion recognition. IEEE Trans. Affect Comput. 13, 568–578. 10.1109/taffc.2019.2922912

[B95] Yuvaraj R. ; Thagavel P. Thomas J. Fogarty J. Ali F. Guo Y. (2023). Comprehensive analysis of feature extraction methods for emotion recognition from multichannel EEG recordings. Sensors 23 (23), 915. 10.3390/s23020915 36679710 PMC9867328

[B96] Zhang B. Shang P. Mao X. Liu J. (2023). Dispersion heterogeneous recurrence analysis and its use on fault detection. Commun. Nonlinear Sci. Numer. Simul. 117, 106902. 10.1016/j.cnsns.2022.106902

[B98] Zheng W. L. Liu W. Lu Y. Lu B. L. Cichocki A. (2019b). EmotionMeter: a multimodal framework for recognizing human emotions. IEEE Trans. Cybern. 49, 1110–1122. 10.1109/TCYB.2018.2797176 29994384

[B99] Zheng W. L. Zhu J. Y. Lu B. L. (2019a). Identifying stable patterns over time for emotion recognition from eeg. IEEE Trans. Affect Comput. 10, 417–429. 10.1109/taffc.2017.2712143

[B100] Zhong P. Wang D. Miao C. (2019). EEG-based emotion recognition using regularized graph neural networks. Available at: http://arxiv.org/abs/1907.07835.

